# Stability trends in carbocation intermediates stemming from germacrene A and hedycaryol

**DOI:** 10.3762/bjoc.20.101

**Published:** 2024-05-23

**Authors:** Naziha Tarannam, Prashant Kumar Gupta, Shani Zev, Dan Thomas Major

**Affiliations:** 1 Department of Chemistry and Institute of Nanotechnology and Advanced Materials, Bar-Ilan University, Ramat-Gan 52900, Israel

**Keywords:** carbocation, germacrene A, hedycaryol, stability trend, terpenes

## Abstract

In the current work, we analyzed the origin of difference in stabilities among the germacrene A and hedycaryol-derived carbocations. This study focused on twelve hydrocarbons derived from germacrene A and twelve from hedycaryol, which can be divided into three groups: four molecules containing 6-6 bicyclic rings, four 5-7 bicyclic compounds with the carbocation being on the seven-membered ring and the remaining four 5-7 bicyclic compounds with the carbocation on the five-membered ring. The variations in energy within the groups of carbocations (i.e., 6-6 and two kinds of 5-7 bicyclic carbocations) can be ascribed to intramolecular repulsion interactions, as seen from non-covalent interactions plots. Despite the structural similarities between germacrene A and hedycaryol cations, they possess a somewhat different stability trend. These differences are attributed to C^+^···OH intramolecular interactions present in some hedycaryol cations, which are absent in the carbocations derived from germecrene A.

## Introduction

Terpenoids form a large and highly diverse group of natural products with a wide range of usage in the pharmaceutical, cosmetic, agricultural, food, and energy industry. Among their several significant processes, terpenoids play an indispensable role in cell-wall and membrane biosynthesis, sensing, plant defense, electron transport, or conversion of light into chemical energy [1–2]. Based on the number of hydrocarbon units, terpenes are classified into various families like monoterpenes (C10), sesquiterpenes (C15), and diterpenes (C20). Enzymes such as monoterpene, sesquiterpene, and diterpene synthases act on geranyl diphosphate (GPP), farnesyl diphosphate (FPP), and geranylgeranyl diphosphate (GGPP) to yield mono-, sesqui-, and diterpenes, respectively. These linear precursors (GPP, FPP, GGPP) undergo highly complex cyclisation cascades forming terpenes and terpenoids that often have great structural complexity. For class I terpene synthases this multistep process is initiated by a heterolytic C–O bond cleavage, separating the diphosphate and isoprenoid ion pairs [3–4]. The isoprenoid allylic carbocation has the capability to engage in standard carbocation reactions, including cyclization via intramolecular olefin attack at the positively charged center, Wagner–Meerwein rearrangements, and hydride or proton shifts. This sequence concludes either through deprotonation, resulting in a terpene hydrocarbon, or through nucleophilic water attack, yielding a terpene alcohol [5].

To date, about 80,000 terpenes and terpenoids have been discovered [3], approx. 10% of which are sesquiterpenes, composed of 15-carbon skeletons [6–7]. Sesquiterpenes are mainly distributed in plants and microbes, in the form of alcohols, ketones, lactones, and glycosides, and of these various forms, the oxo derivatives in particular have strong aroma and biological activity. Interestingly, in addition to being used routinely as flavorings and aromatic agents, sesquiterpene oxo derivatives also have anticancer, antimalarial, antibacterial, and antiviral activity [8–9]. For instance, the well-known artemisinin family of drugs, which is currently the first line of treatment against malaria, is a sesquiterpene lactone [10]. Sesquiterpenes produced by plants [10] also have plant growth regulating and insecticidal activities [11], and are bio-fuel alternatives [12–13].

**Scheme 1:**
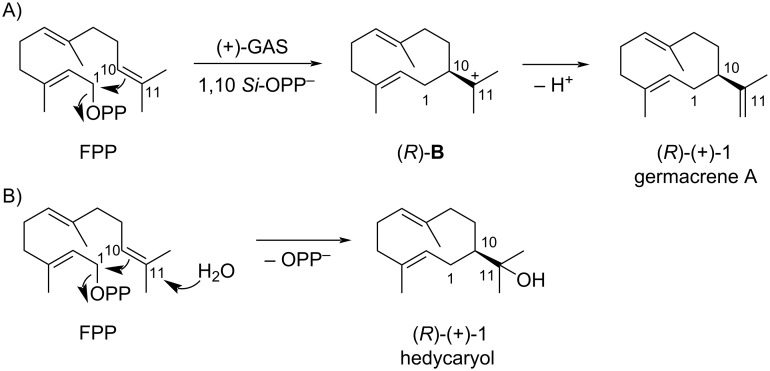
Biosynthesis of (A) germacrene A and (B) hedycaryol from FPP. Here the abbreviations represent, FPP = farnesyl diphosphate, GAS = germacrene A synthase, OPP = diphosphate.

Sesquiterpene synthase can convert FPP to various terpenoids via different initial cyclization processes: 1,6-cyclization to yield the bisabolyl cation, 1,7-cyclization to form the cycloheptanyl cation, 1,10-cyclization lead to the germacradienyl cation, and 1,11-cyclization resulting in the humulyl cation [14–15]. Deprotonation of the intermediate germacradienyl cation yields germacrene A, a doorway towards the synthesis of many eudesmane and guaiane sesquiterpene hydrocarbons through its reprotonation-induced transannular reactions (Scheme 1) [16]. As an alternative to deprotonation, the germacradienyl cation can be captured by water to yield the sesquiterpene alcohol hedycaryol, which is an important intermediate towards the synthesis of sesquiterpene alcohols [17].

**Figure 1:**
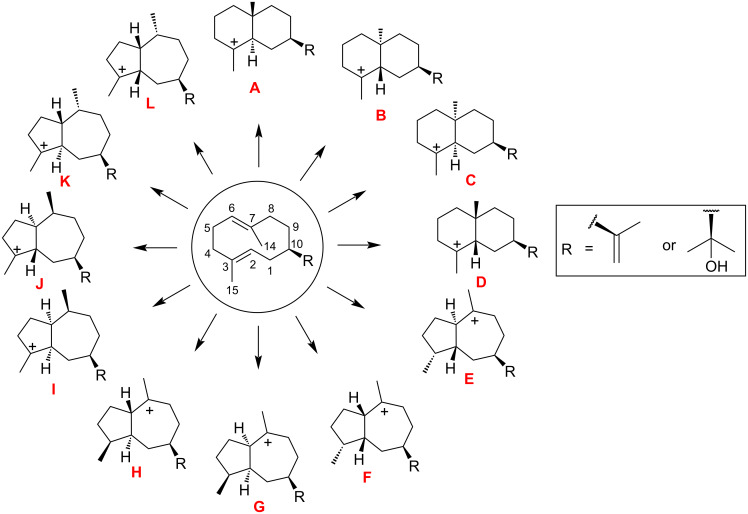
6-6 and 5-7 bicyclic carbocations formed by protonation and cyclization of germacrene A and hedycaryol.

The reprotonation of (*R*)-(+)-1 germacrene A or hedycaryol at the C6 position of the C6–C7 double bond leads to 4 distinct 6-6 bicyclic cationic stereoisomers (**A**–**D**, Figure 1). Reprotonation at the C3-position of the C2–C3 double bond forms 6-6 bicyclic compounds that are not observed in nature for eudesmanes as they proceed via a secondary carbocation [16]. Furthermore, (*R*)-(+)-1 germacrene A or hedycaryol can be protonated at C7, leading to cyclization and formation of a 5-7 bicyclic skeleton (**I**–**L**), which are precursors to guaiane sesquiterpenes. Alternatively, guaiane precursors can be formed by protonation at C3 resulting in **E**–**H**. These carbocations derived from germacrene A or hedycaryol are categorized depending upon the formation of (6,6) or (5,7) cyclic rings. Deprotonations of **A** at the C3 and C15 positions lead to α-selinene [18] and β-selinene [19]. Sesquiterpenes arising through **B** occur less frequently in nature compared to the **A** derivatives, whereas natural products from **C** are unknown and a few sesquiterpenes are known products of **D**. Similarly, in the case of hedycaryols, cation **A-OH** can undergo deprotonations to yield α-eudesmol [20], β-eudesmol [14], or γ-eudesmol [15]. Cation **B-OH** can potentially lead to alcohols by deprotonation or to diols by addition of water.

For each of these intermediates, simple deprotonation or nucleophilic attack by water are possible. Also, hydride shifts can occur first, which widens the chemical space of possible products. Additionally, the presence of multiple stereocenters adds to the rich stereochemistry.

Theory is an important tool in understanding the complex chemistry in terpene synthesis. Gas-phase and in-enzyme tools have been employed extensively to understand terpene chemistry in general and terpene synthases in particular [21–29]. In the current work, we analyze the intermediates formed from germacrene A and hedycaryol. These carbocations are key mechanistic branching points in different sesquiterpene synthases and calculation of their relative energy trends can explain the preference for (6,6) or (5,7) intermediate carbocations. Considering that the formation of (6,6) vs (5,7) is rooted in very slight changes in mechanism (protonation at C1 vs C10), it is of interest to understand whether there is a systematic difference in energy. In cases where enzymes use pathways with high-energy intermediates, the enzyme active site must in some way direct the reaction trajectory towards this pathway and make sure that it stays along this pathway and does not rearrange to a similar, more stable carbocation. Here, using density functional theory (DFT) calculations, we provide a rationale for the relative stability of the intermediate carbocations formed from germacrene A and hedycaryol and how this might affect product distribution in chemical synthesis and biosynthesis. Additionally, we compare the effect of the choice of DFT functional and basis set on the results.

## Methods

The structures of all studied intermediate carbocations originating from germacrene A and hedycaryol were prepared from their corresponding SMILES string using the RDKit library [30]. Electronic structure calculations on these carbocations were performed using the hybrid DFT functional M06-2X [31] and with the range-separated hybrid meta-GGA functional ωB97M-V [32] with the 6-31+G(d,p) basis set [33]. Additionally, to check the reliability of the energy calculations single point calculations were also performed with the larger def2-TZVPP [34] basis set for both functionals and to account for intramolecular non-covalent interactions, D3 dispersion corrections were added to the M06-2X calculations with zero damping [35]. Extensive benchmarking on different sets of classical and non-classical carbocations have shown that M06-2X in conjunction with the 6-31+G(d,p) basis set performs well for carbocations [29,36], and here we confirm this finding for hydroxylated carbocations. All gas-phase calculations were performed using Gaussian 16 (revision A.03) [37] and Q-Chem (version 5.4.2) [38], with default tight geometry optimization convergence criteria (10^−5^ au) and SCF convergence thresholds (10^−8^ au). All stationary points were characterized by frequency calculations. Throughout this article, only the minimum geometries are reported, as no transition states were obtained because the carbocation ring formation is a spontaneous process. Moreover, it is noteworthy that for each cation optimized, we examined several other conformers (specifically rotamers), but these were of higher energy than the ones presented here, and therefore were neglected.

We used the non-covalent interactions (NCI) NCIplot analysis with the program NCIPLOT [39–41] to study the non-covalent interactions present in these molecules. To map local binding properties with this method, two scalar fields are used: the electron density (ρ) and the reduced-density gradient (RDG, *s*).




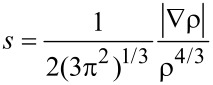




The NCIplot provides qualitative information, and it can successfully map real-space regions where non-covalent interactions are prominent. The resulting plots have a color scheme of red–green–blue scale, where red represents attractive interactions and blue represents repulsive interactions.

Additionally, we carried out natural bonding orbital (NBO) analysis using the NBO 3.1 program [42] as implemented in the Gaussian 16 package. The computations are performed at the same level of theory that we chose initially for optimization (M06-2X/6-31+G(d,p)). This analysis provides insight to the strength of various types of charge transfers usually expressed in the form of second order perturbation energy (*E(2)*)*.*

## Results and Discussion

**Figure 2:**
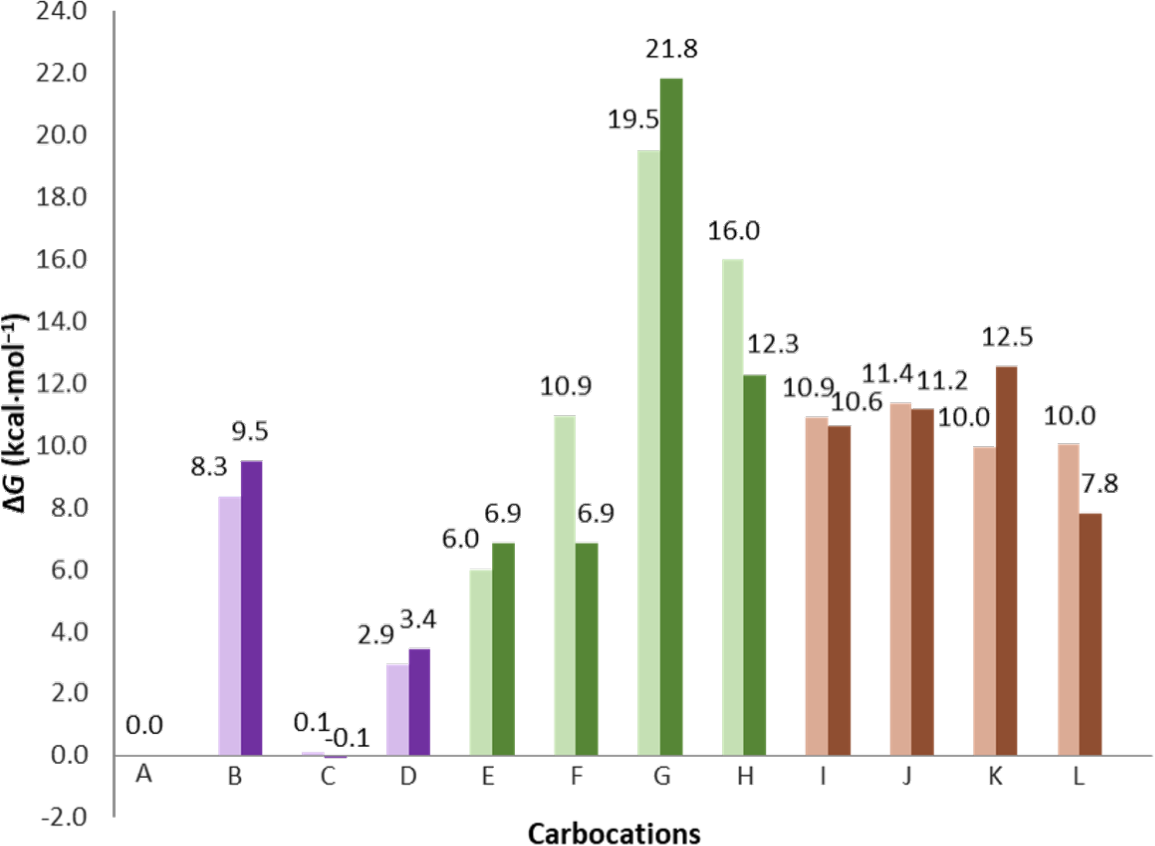
Bar plot for the relative free energies of germacrene A and hedycaryol carbocations relative to carbocation A. Germacrene A carbocations are shown in light color shades and hedycaryol carbocations are represented by dark shades. 6-6 bicyclic molecules are colored purple, 5-7 bicyclic molecules with the carbocation present on the seven membered ring in green, and 5-7 bicyclic molecules with the carbocation on the five membered ring in brown.

The twelve hydrocarbons derived from germacrene A can be divided into three groups: four molecules containing 6-6 bicyclic rings, four 5-7 bicyclic compounds with the carbocation located on the seven-membered ring and the remaining four 5-7 bicyclic compounds with the carbocation on the five-membered ring. 4-8 Bicyclic rings were not considered. Similar to germacrene, the hedycaryol carbocations can also be divided into three analogous sets. Below we will describe the free energy trends for the germacrene A and hedycaryol carbocations (Figure 2), while the corresponding electronic energy trends are presented in Supporting Information File 1 (Figure S1).

### Gibbs free energy of germacrene **A**–**L** carbocations

For the germacrene cations, among the first set of compounds, i.e., 6-6 bicyclic molecules (Figure 2 and Figure S1 in Supporting Information File 1), **A** and **C** are most stable, **D** is less stable by 2.9 kcal/mol, while **B** is the least stable (8.3 kcal/mol). In the case of the 5-7 bicycles with a seven-membered ring carbocation, **E** and **F** are 6.0 and 10.9 kcal/mol less stable than **A**, **H** is 16.0 kcal/mol above **A**, whereas **G** is the least stable (19.5 kcal/mol). For the remaining set (**I**–**L**), the free energy variation is smaller, with values ranging from 10.0 to 11.4 kcal/mol relative to **A**.

**Figure 3:**
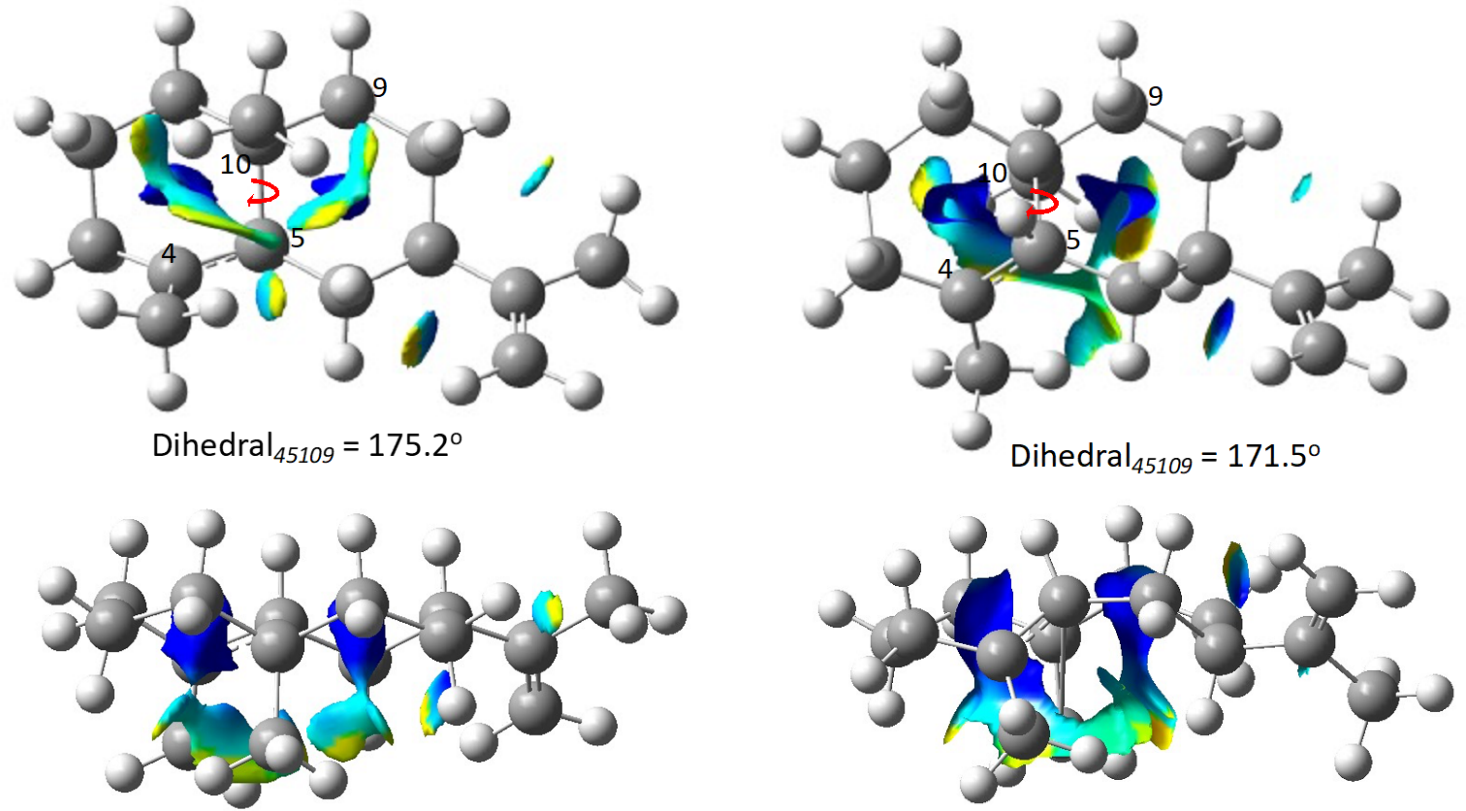
NCI plot for **A** (left) and **B** (right). Blue corresponds to repulsive and yellow represents slightly attractive interactions. The bottom two plots are side views of the cations and emphasize the relatively puckered structure of **B** compared to **A**.

To understand the reason for the difference in free energy, we analyzed the NCI plots for germacrene cations **A**–**D**. Despite being isomers, these carbocations possess quite different geometric arrangements. For instance, molecule **A** is more planar than the puckered **B**. It is clear from visual inspection of the NCI plots that **B**, with a larger blue isosurface than **A**, has greater steric hindrance than **A** (Figure 3). Although this difference is not directly quantified here, this is likely part of the reason behind the greater stability of **A** over **B**. A similar analysis can be performed for **C** and **D** (Figure S2 in Supporting Information File 1).

The NCI plot analysis for the other two sets of carbocations **E**–**L** are presented in Supporting Information File 1 (Figure S2). Among them **G** has the highest Gibbs free energy. Inspection of its geometry and corresponding NCI plot reveals that it has the most puckered structure of all the carbocations with the highest number of H–H Pauli repulsive interactions. This is likely the reason behind carbocation **G**'s instability.

### Gibbs free energy of hedycaryol **A-OH**–**L-OH** carbocations

**Figure 4:**
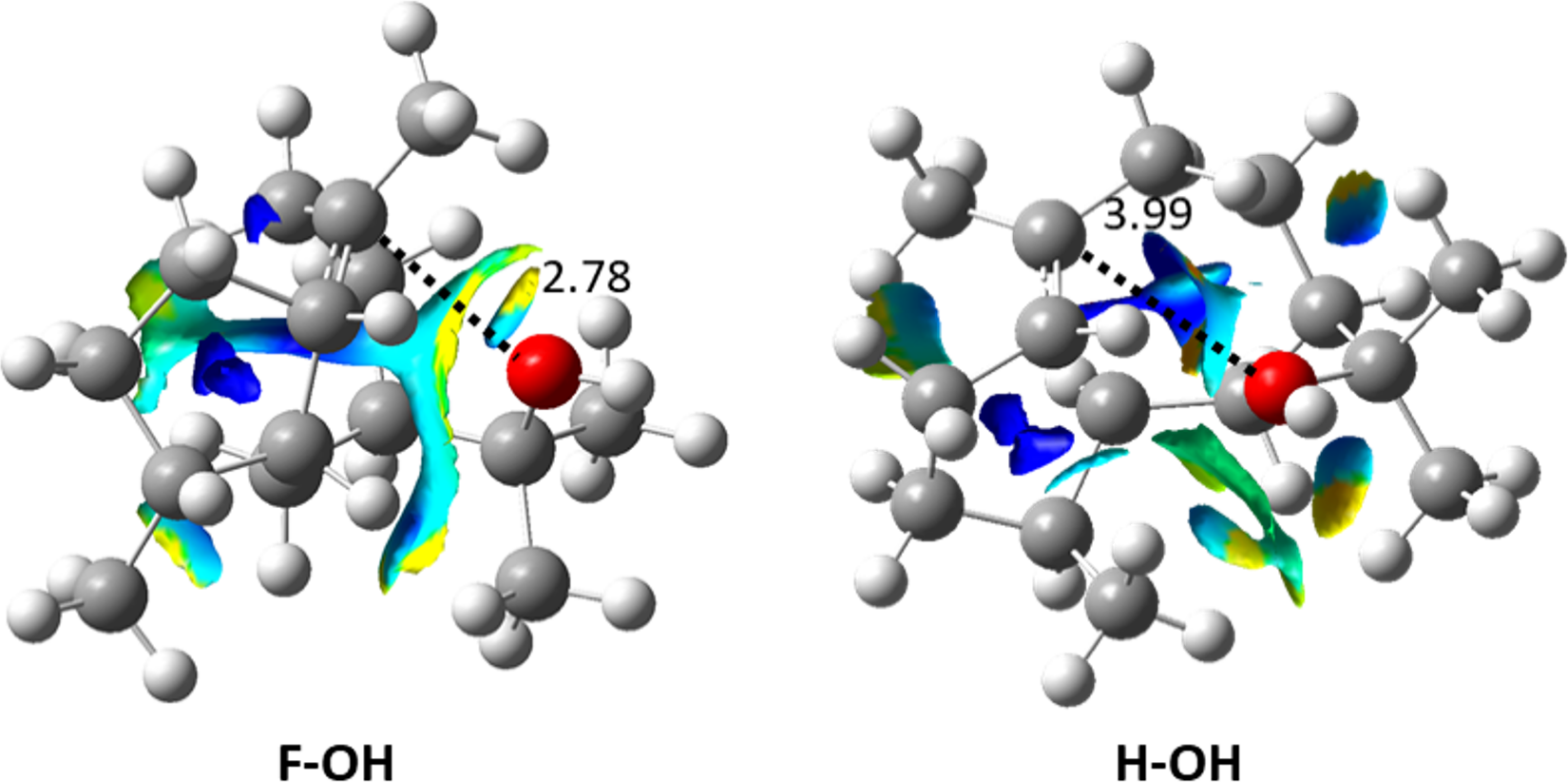
NCI plots for **F** and **H** hedycaryol cations. The C^+^···OH distances (Å) are shown in black.

The hedycaryol cations follow similar free energy trends to the germacrene cations (shown in the dark shades in Figure 2 and Figure S2 in Supporting Information File 1), e.g., carbocations **A-OH** and **C-OH** are more stable than **B-OH** and **D-OH**, and this can be explained by the more sterically hindered geometries of **B-OH** and **D-OH** compared to that of **A-OH** and **C-OH**. However, when inspecting the Gibbs free energy of **F-OH** and **H-OH** (Figure 2), we see that these carbocations are relatively more stable than in the case for **F** and **H**. The break in trend between germacrene A and hedycaryol carbocations is due to the nearby hydroxy group’s ability to stabilize carbocations (i.e., **F-OH** and **H-OH**). The effect of this intramolecular interaction is even more distinct when inspecting the electronic energies (Figure S1, Supporting Information File 1), where we observe a significant stabilizing effect of the hydroxyl–cation interaction for **F-OH** and **H-OH**. This is clearly discernible from the C^+^···OH distances (Figure 4, Table 1) and visible from the NCI plots. In general, stabilization of some 5-7 bicyclic carbocations (e.g., **F-OH** and **H-OH**) are via C^+^···OH interactions, which are facilitated by the carbocation and hydroxyl functionality being located in the relatively flexible 7-membered ring. This type of interaction is absent in case of the 6-6 bicyclic rings. Inspection of the two NCI plots in Figure 4 shows that hedycaryol **F-OH** has the larger isosurface with the most significant attractive region (yellow) among the two. This is supported by the fact that in **F-OH**, the C^+^···OH bond distance is the shortest (Figure 4, Table 1).

**Table 1:** O–C(cation) bond lengths (Å) in the hedycaryol cations.

Molecule	O–C^+^ bond length (Å)

**A-OH**	5.12
**B-OH**	5.39
**C-OH**	4.86
**D-OH**	5.24
**E-OH**	4.12
**F-OH**	2.78
**G-OH**	4.88
**H-OH**	3.99
**I-OH**	5.03
**J-OH**	4.75
**K-OH**	5.08
**L-OH**	4.06

It is noteworthy that optimization with ωB97M-V/6-31+G(d,p) and single point calculations performed with M06-2X and ωB97M-V using the triple-ζ def2-TZVPP basis set provide similar trends for both germacrene and hedycaryol carbocations (the energy comparison plots for the different methods are added in Supporting Information File 1 (Figure S4)). This is evidence that our original method of choice is reliable for this type of studies and in agreement with our earlier benchmark study [36]. Additionally, we carried out single point energy calculations in solvent (chloroform) [43–44] using the SMD [45] solvation model. However, the there was no significant change in the trend in energies for both series of carbocations (Figure S5, Supporting Information File 1).

### NBO analysis

The charge transfer between filled Lewis-type NBOs and empty non-Lewis-type NBOs can provide information regarding the relative stabilities of the carbocations. We note that the second order perturbation (*E*(2)) energies correspond to interactions between the oxygen's lone pair and the nearby antibonding orbitals of C^+^.

As seen in Table 1, the C^+^···OH bond length is the shortest in **F-OH** followed by **H-OH**. NBO analysis provides a clear understanding of the stability trend between **F-OH** and **H-OH**. The second order perturbation energy (*E*(2)) value for O(LP) to C(LV) (LP = lone pair, LV = lone valence orbitals) charge transfer in **F-OH** is 2.9 kcal/mol, whereas there is no such charge transfer in **H-OH**. This explains the relative stability of **F-OH** compared to **H-OH**. Additionally, we carried out NBO analysis for other hedycaryol cations, and due to large bond distances between C^+^···OH, no such charge transfer is observed, i.e., *E*(2) = 0. In these cases, the interaction is likely of a classical electrostatic nature.

**Figure 5:**
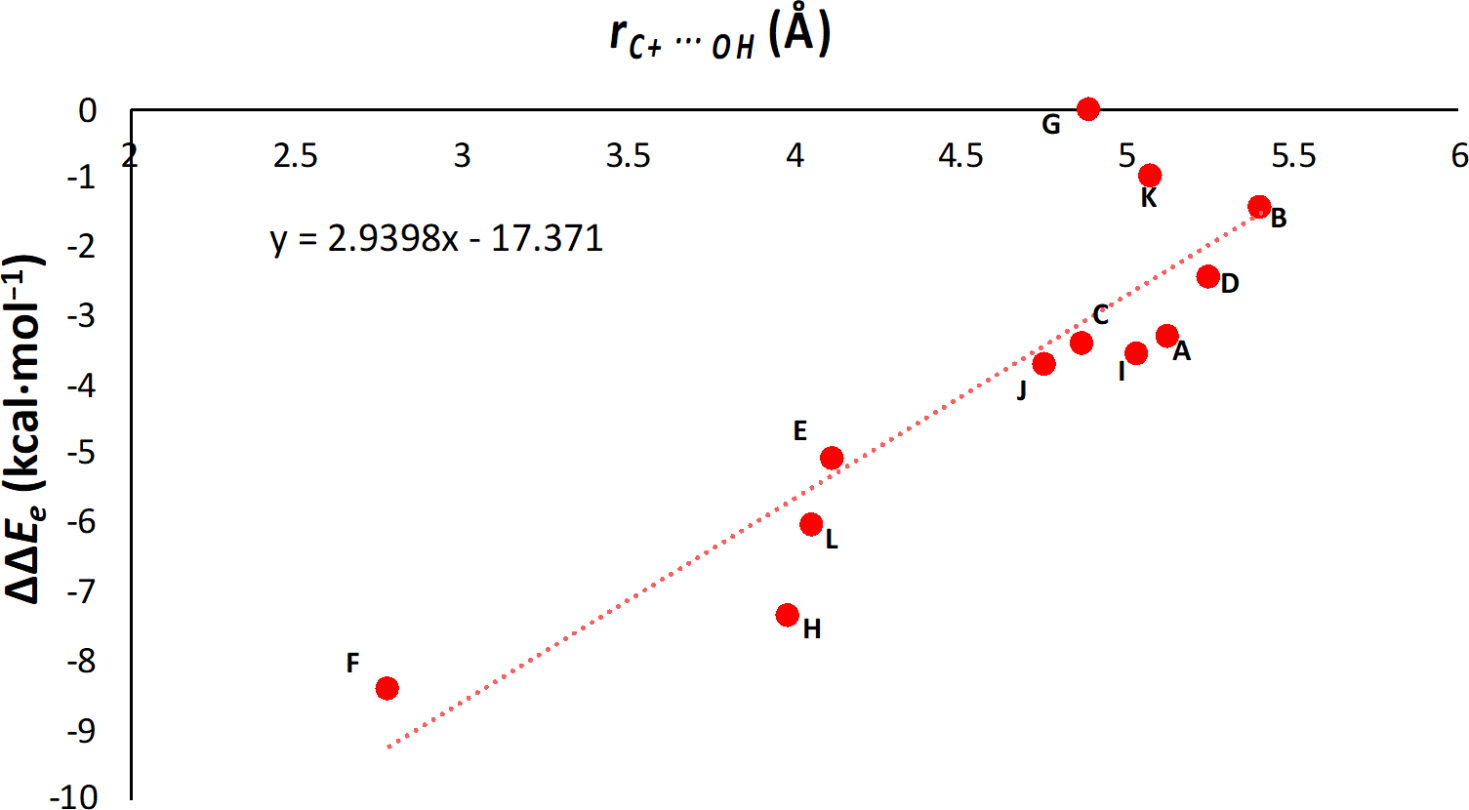
Correlation plot relating between the stability of hedycaryols (ΔΔ*E*_e_) and C^+^···OH bond distances.

Next, to better understand the influence of carbocation and hydroxyl interactions on the stability of hedycaryols, we plotted the C^+^···OH distances with respect to difference in electronic energy for the hedycaryol cations (ΔΔ*E*_e_) (Figure 5). (ΔΔ*E*_e_) is the electronic energy difference between germacrene and hedycaryol cations as presented in the equation below. This analysis allows us to understand the correlation between the stability of hedycaryols and the C^+^···OH distances, as this descriptor presents the difference in stability trends between hedycaryol and germacrene A cations, which is due to the hydroxy group in hedycaryol cations.




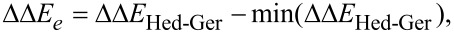




where ΔΔ*E*_Hed-Ger_ = Δ*E*_Hed_ − Δ*E*_Ger_ is the difference between corresponding hedycaryol and germacrene A cations (e.g., **A** and **A-OH**). The correlation coefficient, *R*^2^, is 0.75, indicating a correlation between the energy difference between carbocations and the C^+^-OH distance in hedycaryol cations. For example, **F-OH** has the lowest ΔΔ*E_e_* and the shortest C^+^···OH distance, which agrees with the NCI plots shown above (Figure 4).

An additional peculiar finding is that the standard deviation in free energy and electronic energy follows a distinct pattern. That is, the change in standard deviation follows a trend. When moving in the order of increasing ring size (i.e., 5 to 6 to 7), we observe an increase in the standard deviation (variation in the difference in energy among the different stereoisomers). In case of the 6 membered (**A**–**D**), 7 membered (**E**–**H**) and 5 membered ring (**I**–**L**) cations, the standard deviations in energy and free energy are 0.006, 0.007, 0.002 and 0.006, 0.009, 0.001 kcal/mol, respectively, for germacrenyls. Similarly, for hedycaryolyls 0.007, 0.012, 0.004 and 0.007, 0.011, 0.003 kcal/mol are the respective standard deviations in energy and Gibbs free energy. Examining these values suggests that the three groups of carbocations namely: 6,6-membered rings, 7,5-membered ring with the cation on the 7-membered ring and 7,5-membered ring with the cation on the 5-membered ring obey a trend related to the flexibility of the ring hosting the carbocation.

## Conclusion

In the present work we studied the relative stability of carbocations resulting from protonation and ring closure of germacrene A and hedycaryol. The ring closures considered were: four molecules containing 6-6 bicyclic rings, four 5-7 bicyclic compounds with the carbocation located on the seven-membered ring and the remaining four 5-7 bicyclic compounds with the carbocation on the five-membered ring. The variations in energy within the groups of carbocations (i.e., 6-6 and two kinds of 5-7 bicyclic carbocations) can be ascribed to intramolecular repulsion interactions, as seen from NCI plots. Overall, the 6-6 bicyclic carbocations were more stable than the 5-7 bicyclic compounds. Although the stability trends among the germacrene A and hedycaryol derived cations are similar, some changes in these trends may be ascribed to the hydroxy group in hedycaryol carbocations, which can stabilize the cations via lone pair–cation interaction. Interestingly, enzymes which catalyze reactions proceeding via these intermediates must contend with these intrinsic stability tendencies [24–25,27–28,46–51]. We further found that the M06-2X functional in conjunction with a modest split valence basis set provides rather accurate energies.

## Supporting Information

File 1 Additional figures and Cartesian coordinates for germacrene A and hedycaryol cations.
