# Supplementary material for: Stability trends in carbocation intermediates stemming from germacrene A and hedycaryol
Source: Beilstein J Org Chem. 2024 May 23;20:1189–97. doi: 10.3762/bjoc.20.101 (PMC11181226; doi:10.3762/bjoc.20.101)
Supplement: File 1 — Additional figures and Cartesian coordinates for germacrene A and hedycaryol cations. [file Beilstein_J_Org_Chem-20-1189-s001.pdf]

BEILSTEIN JOURNAL OF ORGANIC CHEMISTRY

Supporting Information

for

Stability trends in carbocation intermediates stemming from germacrene A and hedycaryol

Naziha Tarannam, Prashant Kumar Gupta, Shani Zev and Dan Thomas Major

Beilstein J. Org. Chem. **2024,** *20,* 1189–1197. doi:10.3762/bjoc.20.101

Additional figures and Cartesian coordinates for germacrene A and hedycaryol cations

License and Terms: This is a supporting information file under the terms of the Creative Commons Attribution License (https://creativecommons.org/licenses/by/4.0). Please note that the reuse, redistribution and reproduction in particular requires that the author(s) and source are credited and that individual graphics may be subject to special legal provisions.
The license is subject to the *Beilstein Journal of Organic Chemistry* terms and conditions: (https://www.beilstein-journals.org/bjoc/terms)

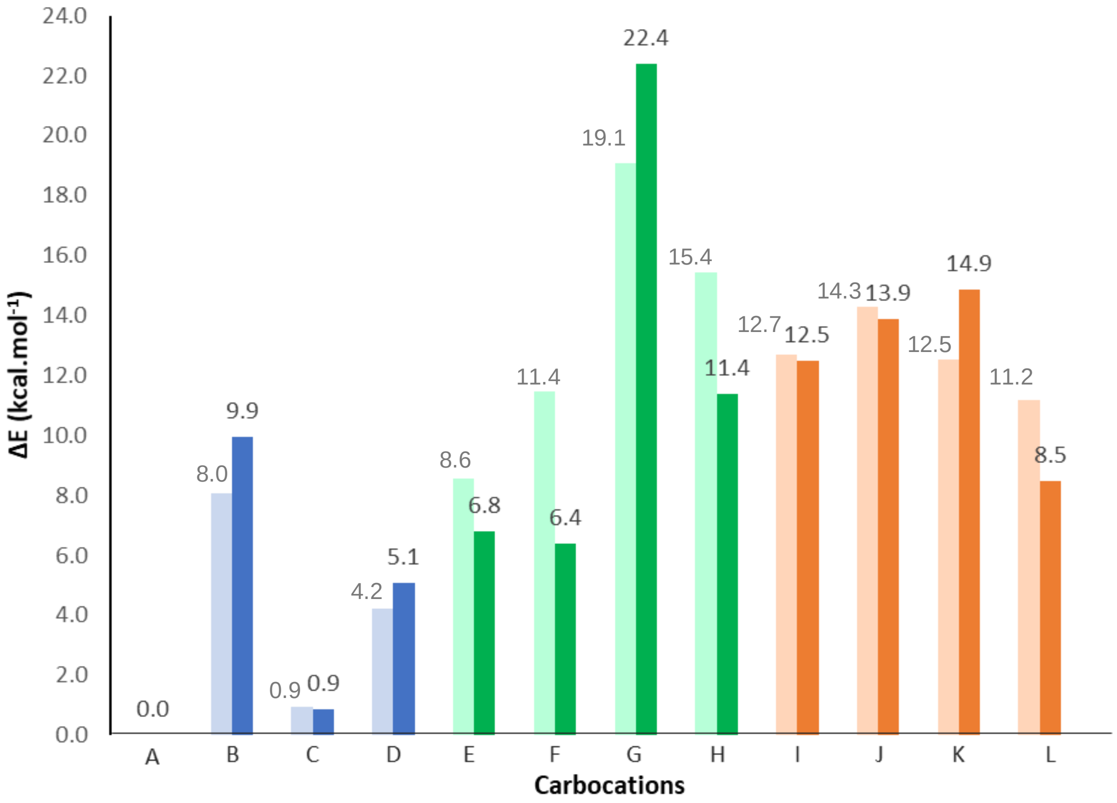

Figure S1: Relative electronic energy bar plots for germacrene A and hedycaryol carbocations with respect to **A** and **A-OH** cations, respectively. Germacrene A carbocations are shown in light color shades and hedycaryol carbocations are represented by dark shades. 6-6 bicyclic molecules are colored in blue; 5-7 bicyclics with the carbocation present on the seven membered ring in green, and 5-7 bicyclics with the carbocation on the five membered ring in orange.

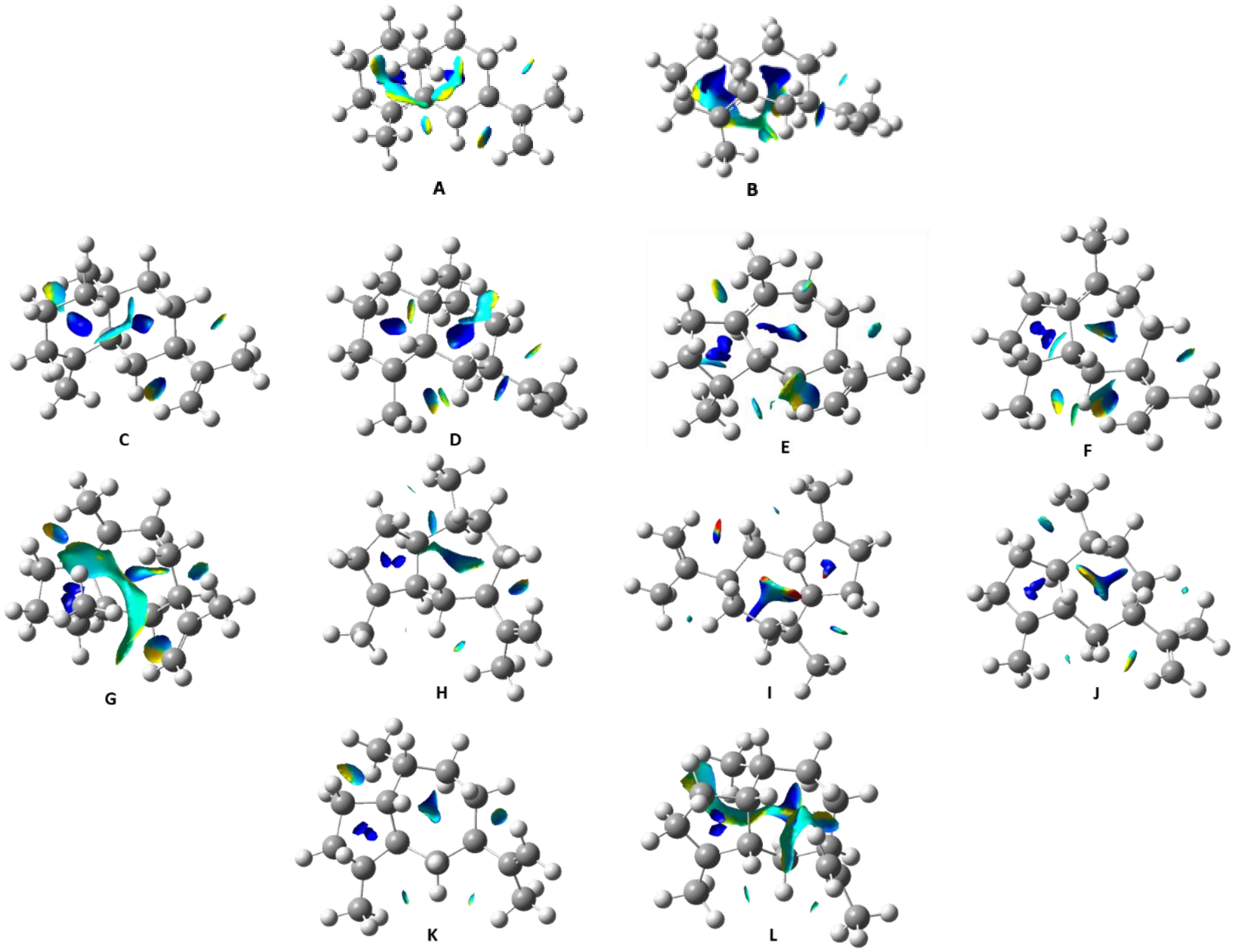

Figure S2: NCI plots for carbocations derived from germacrene A.

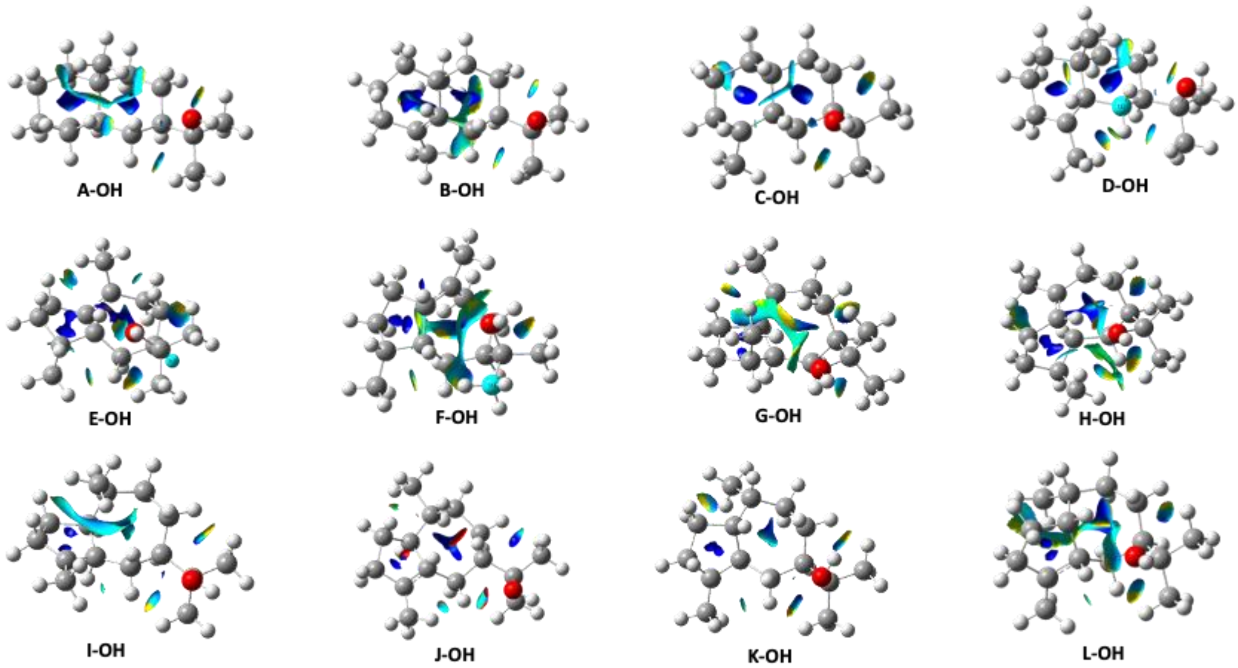

Figure S3: NCI plots for carbocations derived from hedycaryol.

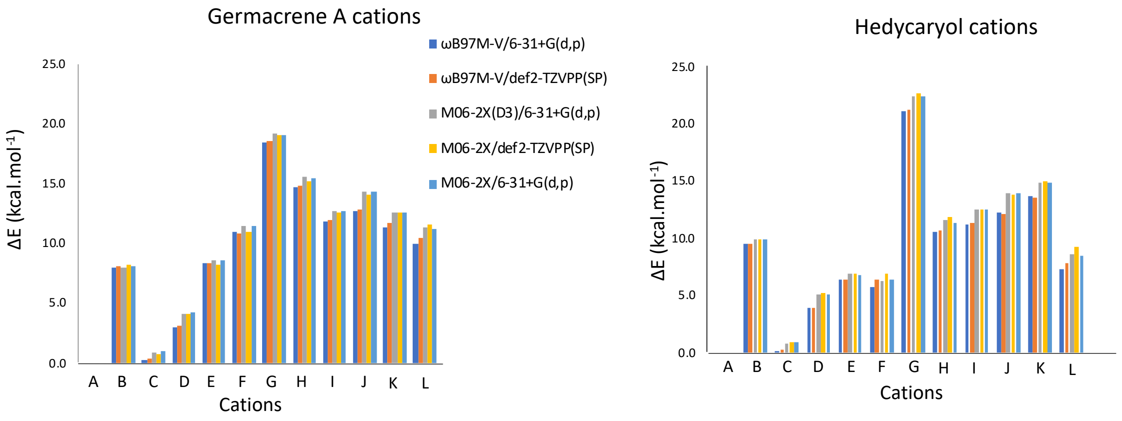

Figure S4: Comparison of energy trends with five different methods tested in this study for both germacrene A and hedycaryol carbocations.

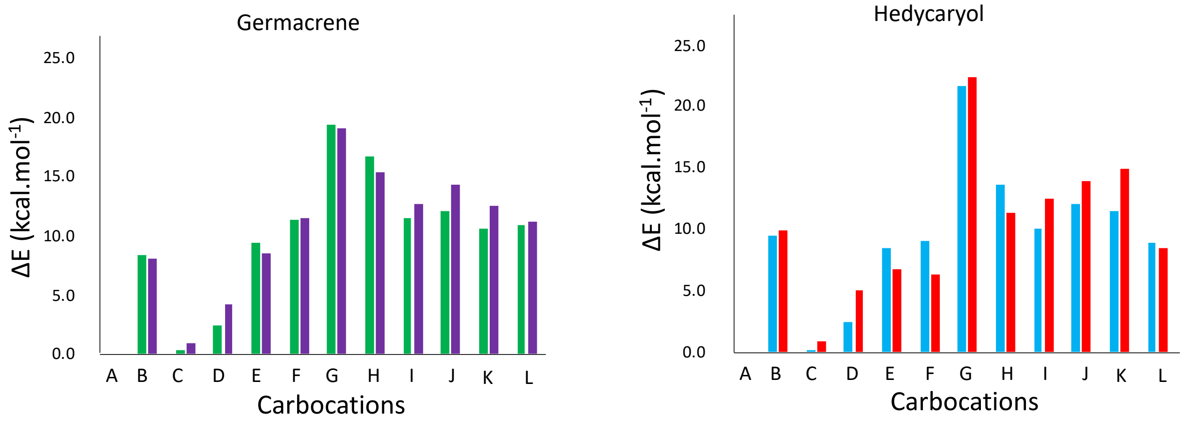

Figure S5: Relative electronic energy bar plots for germacrene A (left) and hedycaryol (right) carbocations with respect to **A** and **A-OH** cations, respectively using chloroform as a continuum solvent. Color codes: green, blue are in solvent, and purple, red are in the gas phase.

Cartesian coordinates for germacrene A and hedycaryol cations optimized at the M06-2X/6-31+G(d,p) level of theory.

Germacrene A carbocations:

A

C	1.8250200000	2.3048980000	0.6315290000
C	1.7988820000	1.2145750000	-0.3524420000
C	3.0372940000	0.8202530000	-1.0292360000
C	3.4733250000	-0.4952610000	-0.2719210000
C	2.3346490000	-1.5057100000	-0.2893600000
C	1.0204700000	-0.9612120000	0.2839500000
C	1.1335520000	-0.6947980000	1.7876490000
C	0.6287070000	0.4013300000	-0.5729550000
C	-0.7427290000	0.9484970000	-0.1785930000
C	-1.8397230000	-0.0834980000	-0.4487680000
C	-3.2384770000	0.4223600000	-0.1458500000
C	-3.4863510000	1.6123260000	0.4071140000
C	-4.3573630000	-0.5190670000	-0.5154940000
C	-1.5320780000	-1.3821060000	0.3158350000
C	-0.1413080000	-1.9255260000	-0.0132460000
H	2.8264260000	2.4951040000	1.0235190000
H	1.5300090000	3.2008420000	0.0534750000
H	1.0874300000	2.1954590000	1.4273990000
H	3.8219730000	1.5746020000	-0.9504590000
H	2.8573100000	0.5555270000	-2.0747620000
H	4.3571600000	-0.8728740000	-0.7910590000
H	3.7813280000	-0.2445000000	0.7473340000
H	2.1665650000	-1.8495020000	-1.3175230000
H	2.6224240000	-2.3875250000	0.2952480000
H	0.2520050000	-0.1925910000	2.1918000000
H	1.2193110000	-1.6561020000	2.3035230000
H	2.0117050000	-0.1019890000	2.0584160000
H	0.6227340000	0.0285970000	-1.6046690000
H	-0.7636530000	1.2260500000	0.8809390000
H	-0.9403300000	1.8606630000	-0.7535500000
H	-1.8155280000	-0.3235440000	-1.5254290000
H	-2.7066200000	2.3124460000	0.6884470000
H	-4.5060130000	1.9310660000	0.5979960000
H	-4.4019470000	-1.3753890000	0.1657710000
H	-4.2221330000	-0.9149710000	-1.5282680000
H	-5.3226460000	-0.0122490000	-0.4727370000
H	-1.6382720000	-1.1995180000	1.3928200000
H	-2.2669230000	-2.1495310000	0.0588360000
H	0.0480810000	-2.8420730000	0.5591270000
H	-0.1036270000	-2.2003700000	-1.0754700000

B

C	-1.2223420000	2.1085590000	1.1617990000
C	-1.6388030000	1.2958950000	0.0089490000
C	-3.0694400000	1.1689140000	-0.3423620000
C	-3.5293710000	-0.3085390000	-0.0626050000
C	-2.4798880000	-1.3164020000	-0.5254270000
C	-1.0765390000	-0.9627890000	-0.0240980000
C	-1.0314650000	-1.0214130000	1.5085500000
C	0.0268000000	-1.8437850000	-0.6236970000
C	1.4429800000	-1.4965540000	-0.1292480000
C	1.6848690000	0.0187170000	0.0969650000
C	3.1596500000	0.3541950000	-0.0369510000
C	4.0594120000	-0.3833020000	0.9218030000
C	3.6439520000	1.2297630000	-0.9206960000
C	0.7831890000	0.8576070000	-0.8091140000
C	-0.7161790000	0.5198270000	-0.7539400000
H	-0.2272280000	1.8617050000	1.5338560000
H	-1.9688450000	2.1057120000	1.9607810000
H	-1.1851610000	3.1440900000	0.7787370000
H	-3.6931310000	1.8680580000	0.2178610000
H	-3.1845120000	1.3557940000	-1.4157760000
H	-3.7294250000	-0.4157690000	1.0062210000
H	-4.4795250000	-0.4575990000	-0.5790790000
H	-2.4743210000	-1.3964760000	-1.6186600000
H	-2.7425220000	-2.3072240000	-0.1361420000
H	-0.0842420000	-0.6825710000	1.9259240000
H	-1.1537110000	-2.0709550000	1.7961510000
H	-1.8307560000	-0.4594770000	1.9964930000
H	-0.0264470000	-1.7740350000	-1.7164690000
H	-0.2102690000	-2.8835750000	-0.3659430000
H	1.6569560000	-2.0379770000	0.7972810000
H	2.1593540000	-1.8622500000	-0.8722670000
H	1.4326510000	0.2562660000	1.1403570000
H	4.0647350000	-1.4595630000	0.7175650000
H	5.0872520000	-0.0243770000	0.8510920000
H	3.7210780000	-0.2525700000	1.9564620000
H	4.7093500000	1.4304080000	-0.9695600000
H	3.0217310000	1.7745400000	-1.6229000000
H	0.9314200000	1.9224080000	-0.5988480000
H	1.0959480000	0.7056810000	-1.8476820000
H	-1.1113320000	0.3355860000	-1.7550230000

C

C	2.0038810000	2.3767740000	-1.2969570000
C	1.7451690000	1.2545550000	-0.3872220000
C	2.4414050000	1.2087240000	0.9082470000
C	2.6639480000	-0.1873070000	1.5125040000
C	1.4715460000	-1.1140180000	1.2707010000
C	1.0556960000	-1.1750710000	-0.2100190000
C	2.1966770000	-1.7473600000	-1.0630690000
C	0.7520690000	0.2509740000	-0.7283210000
C	-0.5608690000	0.8793970000	-0.0830930000
C	-1.7510860000	-0.0398090000	-0.3790050000
C	-3.0608150000	0.5296320000	0.1426400000
C	-3.1469130000	1.6291400000	0.8954770000
C	-4.2905560000	-0.2504670000	-0.2515250000
C	-1.4802340000	-1.4476910000	0.1741600000
C	-0.1967060000	-2.0438570000	-0.4048540000
H	2.3017420000	3.2908350000	-0.7776670000
H	2.8852560000	2.0457290000	-1.8778960000
H	1.1960050000	2.5494530000	-2.0099040000
H	1.7427770000	1.7865640000	1.5502530000
H	3.3472990000	1.8230880000	0.8756170000
H	3.5744600000	-0.6110310000	1.0794610000
H	2.8535790000	-0.0763310000	2.5822430000
H	0.6273520000	-0.7951920000	1.8920450000
H	1.7289150000	-2.1258560000	1.6012190000
H	1.8980280000	-1.8225150000	-2.1134900000
H	2.4646520000	-2.7510550000	-0.7192820000
H	3.1016000000	-1.1286270000	-1.0214870000
H	0.5741880000	0.2498110000	-1.8124620000
H	-0.4296720000	0.9745840000	0.9996360000
H	-0.7420430000	1.8773240000	-0.4950800000
H	-1.8484500000	-0.1213560000	-1.4735520000
H	-4.1122660000	1.9887410000	1.2365020000
H	-2.2858990000	2.2125150000	1.2049680000
H	-4.3546880000	-1.1972760000	0.2944680000
H	-4.2817790000	-0.4896290000	-1.3205640000
H	-5.1955530000	0.3194020000	-0.0352470000
H	-1.4426480000	-1.3971300000	1.2698160000
H	-2.3147120000	-2.1078500000	-0.0759000000
H	-0.0061450000	-3.0315490000	0.0305140000
H	-0.3412840000	-2.2043520000	-1.4822040000

D

C	0.7574830000	0.3215400000	0.8089930000
C	1.3517140000	1.4774000000	0.1220220000
C	0.7366340000	2.8117500000	0.2279650000
C	2.6421160000	1.3897370000	-0.5748630000
C	3.1187050000	0.0110190000	-1.0255740000
C	2.7260090000	-1.0551860000	-0.0093960000
C	1.2229830000	-1.0776890000	0.3148780000
C	1.0019110000	-2.1177030000	1.4219200000
C	0.3916520000	-1.4290490000	-0.9357440000
C	-1.1210570000	-1.3352170000	-0.7265150000
C	-1.5363690000	0.0603090000	-0.2299090000
C	-3.0410400000	0.1870410000	-0.0824150000
C	-3.8332880000	-0.0249510000	-1.3484480000
C	-3.6436310000	0.4566660000	1.0786850000
C	-0.7626700000	0.4073710000	1.0484250000
H	1.2607630000	0.5055260000	1.7950570000

H	1.4131610000	3.6128680000	-0.0696850000
H	-0.1233240000	2.8068580000	-0.4635110000
H	0.3129160000	2.9966480000	1.2183950000
H	3.3181040000	1.7902220000	0.2137850000
H	2.6894260000	2.1612100000	-1.3534600000
H	2.6981560000	-0.2096650000	-2.0103390000
H	4.2031260000	0.0383030000	-1.1536130000
H	3.2881830000	-0.8973540000	0.9222210000
H	3.0186790000	-2.0429620000	-0.3801880000
H	1.3581800000	-3.0933410000	1.0783920000
H	1.5663620000	-1.8572530000	2.3240490000
H	-0.0462500000	-2.2346650000	1.7013210000
H	0.6587930000	-0.7449320000	-1.7548660000
H	0.6687680000	-2.4361930000	-1.2678840000
H	-1.6204280000	-1.5573040000	-1.6733780000
H	-1.4684940000	-2.0866470000	-0.0071070000
H	-1.2365400000	0.7764690000	-1.0188220000
H	-3.4014880000	0.5340340000	-2.1865020000
H	-3.8488460000	-1.0812670000	-1.6365470000
H	-4.8674620000	0.2978710000	-1.2189430000
H	-3.1060040000	0.5973240000	2.0105370000
H	-4.7245800000	0.5366730000	1.1335560000
H	-1.0219420000	-0.3031500000	1.8381300000
H	-1.0561640000	1.3925010000	1.4192570000

E

C	-2.2702030000	-1.8062550000	-1.1152440000
C	-2.6975090000	-0.8417440000	-0.2958210000
C	-1.8211220000	-0.1681020000	0.7512940000
C	-0.4568600000	-0.8507670000	0.9665150000
C	0.6360590000	-0.4764660000	-0.0274720000
C	1.7414620000	-1.5236080000	-0.3167170000
C	2.2663360000	-2.2096670000	0.9430330000
C	2.8208160000	-0.7159400000	-1.0417360000
C	2.9665530000	0.5683540000	-0.2021750000
C	1.6203590000	0.7628500000	0.5102810000
C	0.6920120000	1.7671070000	0.1235380000
C	0.8103960000	2.4726840000	-1.1719360000
C	-0.4832150000	2.0768250000	0.9737470000
C	-1.7352400000	1.3414720000	0.4072760000
C	-4.1144710000	-0.3287840000	-0.3602830000
H	-1.2624360000	-2.2094430000	-1.0885320000
H	-2.9394950000	-2.2457600000	-1.8476950000
H	-2.3553210000	-0.2478970000	1.7075150000
H	-0.6164470000	-1.9324430000	0.9090540000
H	-0.1017680000	-0.6673440000	1.9865760000
H	0.1914590000	-0.2091190000	-0.9960920000
H	1.2978350000	-2.2778220000	-0.9792230000
H	1.4825830000	-2.7638860000	1.4647610000
H	2.7053630000	-1.4984850000	1.6514060000
H	3.0486940000	-2.9227830000	0.6713240000
H	2.4846360000	-0.4733870000	-2.0562390000
H	3.7628500000	-1.2613070000	-1.1325470000
H	3.7385380000	0.4535340000	0.5619740000
H	3.2520350000	1.4339750000	-0.8043990000
H	1.6559080000	0.6158290000	1.5909760000
H	1.4422730000	3.3516950000	-0.9629910000
H	1.3245470000	1.8869630000	-1.9358010000
H	-0.1481270000	2.8421730000	-1.5394680000
H	-0.3133820000	1.8052590000	2.0179520000
H	-0.6681870000	3.1552690000	0.9110340000
H	-2.6127440000	1.8574050000	0.8024670000
H	-1.7642290000	1.4765940000	-0.6804080000
H	-4.1544940000	0.7148680000	-0.6916650000
H	-4.7153830000	-0.9193690000	-1.0532530000
H	-4.5869510000	-0.3722660000	0.6274710000

F

C	4.0376510000	-0.4485930000	-0.1177780000
C	2.7903550000	0.3993620000	-0.0558940000
C	1.6272530000	-0.2160050000	0.7092370000
C	0.3796250000	0.6745860000	0.8140690000
C	-0.5003550000	0.7124680000	-0.4416590000
C	-1.5438670000	1.8567600000	-0.4258640000
C	-1.0155350000	3.2576050000	-0.1444890000
C	-2.5927760000	1.3653260000	0.5843300000
C	-2.6540810000	-0.1779180000	0.4618720000
C	-1.4507590000	-0.4900720000	-0.5804540000
C	-1.1258520000	-1.8492160000	-0.2250950000
C	-1.9344920000	-2.9408630000	-0.7945510000
C	-0.0108640000	-2.2170150000	0.6708560000
C	1.3123790000	-1.6073910000	0.1274900000
C	2.7504180000	1.6074530000	-0.6236040000
H	4.8807680000	0.1261980000	-0.5036750000
H	4.3065910000	-0.8239370000	0.8763980000
H	3.9056840000	-1.3190780000	-0.7700700000
H	1.9848880000	-0.3817270000	1.7373770000
H	0.7123570000	1.6859270000	1.0577110000
H	-0.2224170000	0.3623650000	1.6773130000
H	0.1344730000	0.7816110000	-1.3331600000
H	-2.0101510000	1.8691900000	-1.4207830000
H	-0.2295060000	3.5343560000	-0.8533930000
H	-0.6173910000	3.3500920000	0.8699380000
H	-1.8207210000	3.9899970000	-0.2458690000
H	-3.5778120000	1.8012930000	0.4007670000
H	-2.2940120000	1.6425090000	1.5999110000
H	-2.4987330000	-0.6636730000	1.4297290000
H	-3.5897040000	-0.5522740000	0.0449800000
H	-1.9344920000	-0.4756500000	-1.5637780000
H	-2.8103660000	-2.6021890000	-1.3471760000
H	-1.2630050000	-3.4736140000	-1.4898140000
H	-2.1979520000	-3.6769460000	-0.0272850000
H	-0.2047350000	-1.8164990000	1.6756430000
H	0.0459610000	-3.3036850000	0.7625320000
H	2.1106600000	-2.3119820000	0.3720880000
H	1.2780760000	-1.5554820000	-0.9690130000
H	1.8772650000	2.2508400000	-0.5922800000
H	3.6196730000	2.0034150000	-1.1388860000

G

C	2.7241270000	-1.7480730000	0.1188550000
C	2.7151320000	-0.4155800000	0.0432010000
C	1.6917530000	0.3892300000	-0.7476930000
C	0.6477240000	-0.4189050000	-1.5488680000
C	-0.7333200000	-0.7984690000	-1.0021570000
C	-0.9571550000	-1.7443020000	0.1918250000
C	-2.4930190000	-1.8403260000	0.2061480000
C	-3.0013100000	-0.4080020000	-0.1119170000
C	-1.8064010000	0.3553090000	-0.7250990000
C	-1.2783130000	1.5320930000	-0.0319350000
C	-1.8774120000	2.0984720000	1.1892570000
C	-0.1179810000	2.2384200000	-0.5755370000
C	1.0913220000	1.5003760000	0.1298080000
C	3.7889020000	0.4084960000	0.7115140000
H	1.9685130000	-2.3752700000	-0.3428630000
H	3.5096700000	-2.2643530000	0.6614990000
H	2.2838010000	0.9143180000	-1.5130960000
H	1.1229590000	-1.3474190000	-1.8782230000
H	0.4558410000	0.1249820000	-2.4818850000
H	-2.8757230000	-2.2041680000	1.1623820000
H	-2.8324990000	-2.5356700000	-0.5664230000
H	-3.8272750000	-0.4308010000	-0.8258490000
H	-3.3910320000	0.0817610000	0.7815460000
H	-2.7102680000	2.7208730000	0.8119010000
H	-2.3183530000	1.3656510000	1.8628520000
H	-1.1933920000	2.7669470000	1.7145310000
H	-0.0175420000	2.1491510000	-1.6577730000
H	-0.1235280000	3.2891920000	-0.2805080000
H	1.8346300000	2.2742610000	0.3332100000
H	0.7896120000	1.1042640000	1.1064110000
H	3.3944090000	0.9728550000	1.5644780000
H	4.5941930000	-0.2267600000	1.0829120000
C	-0.4295890000	-1.2749700000	1.5449930000
H	-0.6703530000	-2.0190690000	2.3092230000
H	0.6518390000	-1.1321880000	1.5482260000
H	-0.9095810000	-0.3406350000	1.8630230000
H	-0.5091230000	-2.7159500000	-0.0481250000
H	-1.2033560000	-1.3147390000	-1.8484520000
H	-2.0312600000	0.7660790000	-1.7257900000
H	4.2210520000	1.1340720000	0.0124100000

H

C	-2.1543500000	1.7818420000	0.7205940000
C	-2.6171320000	0.6397200000	0.2080340000
C	-1.8240590000	-0.2714910000	-0.7178960000
C	-0.5757390000	0.3673440000	-1.3952130000
C	0.8420980000	0.1862940000	-0.8124120000
C	1.8288240000	1.3988940000	-0.7410700000
C	2.9151090000	0.9404230000	0.2404340000
C	2.1377040000	0.2936710000	1.4002480000
C	0.8881540000	-0.3289370000	0.7724140000
C	0.9404990000	-1.6337720000	0.2093560000
C	2.2099180000	-2.4008800000	0.0894740000
C	-0.2915210000	-2.3317490000	-0.2654140000
C	-1.6079710000	-1.6275030000	0.0221470000
C	-4.0041470000	0.1407850000	0.5267330000
H	-1.1742610000	2.1744090000	0.4810640000
H	-2.7661660000	2.3798960000	1.3881780000
H	-2.4971310000	-0.4863950000	-1.5570390000
H	-0.7748650000	1.4373560000	-1.4859710000
H	-0.5351420000	-0.0059460000	-2.4221750000
H	3.5372680000	1.7734340000	0.5756650000
H	3.5842720000	0.2199480000	-0.2411030000
H	2.7278750000	-0.4430800000	1.9518670000
H	1.8219590000	1.0477090000	2.1238770000
H	3.1042370000	-1.7816550000	0.0751090000
H	2.2540380000	-3.0159420000	1.0014260000
H	2.1982990000	-3.0843160000	-0.7616920000
H	-0.1584150000	-2.5522830000	-1.3347480000
H	-0.2681970000	-3.3193660000	0.2191640000
H	-2.4180960000	-2.3126740000	-0.2388670000
H	-1.6872740000	-1.4765040000	1.1049500000
H	-3.9836140000	-0.8300420000	1.0336880000
H	-4.5377800000	0.8425420000	1.1692330000
C	1.2104080000	2.7184430000	-0.2864180000
H	1.9874460000	3.4873800000	-0.3079260000
H	0.4040310000	3.0601420000	-0.9387170000
H	0.8318630000	2.6657820000	0.7387290000
H	2.2400230000	1.5338980000	-1.7476530000
H	1.3774140000	-0.5276040000	-1.4588160000
H	-0.0711740000	-0.0346540000	1.1999540000
H	-4.5833750000	0.0094580000	-0.3942620000

I

C	-0.7565360000	3.3958880000	0.0479510000
C	-1.2768150000	2.0247370000	0.0083060000
C	-2.5249410000	1.6450500000	-0.6665740000
C	-2.5414420000	0.1109340000	-0.6828850000
C	-1.6843360000	-0.2675770000	0.5450160000
C	-0.6308170000	0.8645600000	0.5914000000
C	0.6172930000	0.6739280000	-0.3852980000
C	1.5190260000	-0.4734690000	0.1560260000
C	2.9690920000	-0.0360670000	0.0202580000
C	3.4399230000	0.9005790000	1.1035170000
C	3.7735620000	-0.4257290000	-0.9717120000
C	1.1874310000	-1.8229200000	-0.4835470000
C	-0.3088650000	-2.1547300000	-0.5837850000
C	-1.1978730000	-1.7312040000	0.5986060000
C	-2.4229250000	-2.6502690000	0.6841150000
H	-0.3766660000	3.6330440000	-0.9585700000
H	0.0445740000	3.5334700000	0.7743740000
H	-1.5726460000	4.1061330000	0.2203670000
H	-2.7226510000	2.1827410000	-1.5995050000
H	-3.2826410000	2.0201130000	0.0548700000
H	-2.0970560000	-0.2499630000	-1.6148340000
H	-3.5551260000	-0.2869610000	-0.6261110000
H	-2.3132790000	-0.1185970000	1.4351960000
H	0.2905270000	0.4530170000	-1.4077780000
H	1.1826070000	1.6089580000	-0.4118030000
H	1.3225800000	-0.5548240000	1.2339900000
H	4.4398620000	1.2862830000	0.8989080000
H	2.7654180000	1.7598050000	1.2182210000
H	3.4614370000	0.3844430000	2.0700610000
H	4.7964240000	-0.0655940000	-1.0232840000
H	3.4654950000	-1.1156190000	-1.7490730000
H	1.6067360000	-1.8681790000	-1.4930140000
H	1.7031700000	-2.5940450000	0.0986120000
H	-0.7196890000	-1.7415960000	-1.5127440000
H	-0.3971210000	-3.2393800000	-0.7027010000
H	-0.6265360000	-1.8508370000	1.5301590000
H	-2.9650560000	-2.6694090000	-0.2679660000
H	-2.1157940000	-3.6749830000	0.9052920000
H	-3.1180120000	-2.3318240000	1.4672520000
H	-0.1872310000	1.0715360000	1.5717110000

J

C	1.6875050000	3.1384230000	0.1889740000
C	1.8770320000	1.6973140000	0.0174020000
C	3.1665490000	1.0441040000	-0.2412000000
C	2.8831920000	-0.4613520000	-0.1367440000
C	1.4163050000	-0.5731970000	-0.5900680000
C	0.8143900000	0.7132640000	0.0452260000
C	-0.6261260000	1.1505560000	-0.2034110000
C	-1.6021730000	-0.0304080000	-0.1482300000
C	-3.0586380000	0.4121480000	-0.1966310000
C	-3.9723180000	-0.5239510000	-0.9430290000
C	-3.5157410000	1.5143530000	0.4016230000
C	-1.3751600000	-0.9422060000	1.0793920000
C	-0.0629130000	-1.7791040000	1.1177170000
C	0.6875340000	-1.8921870000	-0.2153790000
C	1.6518940000	-3.0827670000	-0.2177770000
H	2.6148500000	3.6824960000	0.3632130000
H	0.9441330000	3.3501630000	0.9649910000
H	1.2264720000	3.4977530000	-0.7472940000
H	4.0018830000	1.4640900000	0.3269870000
H	3.3586890000	1.3135020000	-1.3016490000
H	2.9853480000	-0.7785880000	0.9086330000
H	3.5715940000	-1.0561880000	-0.7366930000
H	1.3832260000	-0.4458720000	-1.6825740000
H	-0.9005690000	1.8950980000	0.5514230000
H	-0.7014400000	1.6420350000	-1.1812950000
H	-1.4370920000	-0.6276010000	-1.0535280000
H	-5.0174910000	-0.2252290000	-0.8493020000
H	-3.7133540000	-0.5447950000	-2.0078120000
H	-3.8760700000	-1.5505100000	-0.5708360000
H	-4.5711300000	1.7650930000	0.3698940000
H	-2.8794500000	2.2011730000	0.9514970000
H	-1.4606090000	-0.3237930000	1.9821410000
H	-2.2227050000	-1.6324850000	1.1232180000
H	0.6259440000	-1.3965060000	1.8858070000
H	-0.3093240000	-2.7905540000	1.4540160000
H	-0.0492000000	-2.0892660000	-1.0005080000
H	2.3655970000	-3.0306790000	0.6109630000

H	1.0923570000	-4.0148540000	-0.1101620000
H	2.2156070000	-3.1434940000	-1.1538630000
H	0.9247360000	0.5290690000	1.1555510000

K

C	3.8562740000	-0.9726680000	-0.8509800000
C	3.0931120000	-0.1419710000	0.1476600000
C	3.6018220000	0.1846430000	1.3382010000
C	1.6994570000	0.2912610000	-0.2858930000
C	0.7423880000	-0.8642700000	0.0635230000
C	-0.7553920000	-0.5807030000	-0.2869800000
C	-1.4455360000	0.4888990000	0.5937810000
C	-2.8704090000	-0.0580260000	0.7973960000
C	-2.6598880000	-1.5816640000	0.8040120000
C	-1.5512130000	-1.7998420000	-0.1284380000
C	-1.2406760000	-3.0864790000	-0.7514990000
C	-1.3421450000	1.9381790000	0.0798410000
C	-2.3882320000	2.2893740000	-0.9813140000
C	0.0589470000	2.2636980000	-0.4707230000
C	1.2646820000	1.6591540000	0.2653610000
H	4.0709680000	-0.3848940000	-1.7504860000
H	4.8032090000	-1.3235780000	-0.4374580000
H	3.2835220000	-1.8505700000	-1.1736150000
H	3.0643880000	0.8022840000	2.0515100000
H	4.5921780000	-0.1483820000	1.6323030000
H	1.7122590000	0.3777890000	-1.3834280000
H	1.0826840000	-1.7708300000	-0.4474480000
H	0.7935270000	-1.0544250000	1.1434450000
H	-0.7998530000	-0.3339490000	-1.3678850000
H	-0.9361860000	0.4431380000	1.5668510000
H	-3.3413160000	0.3011550000	1.7138060000
H	-3.5215820000	0.2023830000	-0.0415680000
H	-3.5234660000	-2.2335930000	0.6525550000
H	-2.2074910000	-1.8807380000	1.7760800000
H	-0.7151060000	-3.6745130000	0.0227160000
H	-2.1454830000	-3.6496790000	-0.9929440000
H	-0.5717540000	-2.9998580000	-1.6086250000
H	-1.5184630000	2.5806610000	0.9519260000
H	-3.4043660000	2.3150840000	-0.5803650000
H	-2.1775320000	3.2791300000	-1.3951280000
H	-2.3664320000	1.5803330000	-1.8196070000
H	0.1537190000	3.3538390000	-0.4853560000
H	0.1028730000	1.9584510000	-1.5272550000
H	2.1200210000	2.3311930000	0.1516820000
H	1.0705320000	1.6020610000	1.3446030000

L

C	1.4586570000	2.6532510000	-1.1475440000
C	1.4762730000	1.5747010000	-0.1548210000
C	2.6410750000	1.2480890000	0.6781570000
C	2.1946970000	0.1070160000	1.6053960000
C	0.9532770000	-0.5123370000	0.9221190000
C	0.3706230000	0.6753650000	0.1088190000
C	-0.6207330000	0.4091360000	-1.0238240000
C	-1.8051680000	-0.4851020000	-0.5910850000
C	-2.7063790000	0.3218260000	0.3317040000
C	-3.7488290000	1.1562650000	-0.3664850000
C	-2.5752120000	0.3433660000	1.6639850000
C	-1.3802440000	-1.8782710000	-0.0641510000
C	-0.0306950000	-2.3721140000	-0.6003750000
C	1.2387510000	-1.7619960000	0.0671910000
C	2.3450850000	-1.5494350000	-0.9696150000
H	1.5383050000	2.1651900000	-2.1337840000
H	2.2832190000	3.3564820000	-1.0351850000
H	0.4918620000	3.1671550000	-1.1585190000
H	3.0720720000	2.1392570000	1.1511550000
H	3.4072820000	0.9250090000	-0.0523230000
H	1.9210540000	0.5209490000	2.5800570000
H	2.9885600000	-0.6238540000	1.7732060000
H	0.2333970000	-0.7956090000	1.6906610000
H	-0.1668580000	1.3185610000	0.8658540000
H	-1.0168460000	1.3591870000	-1.3995660000
H	-0.0844560000	-0.0606180000	-1.8551760000
H	-2.3669450000	-0.6529680000	-1.5193800000
H	-3.3117330000	1.7634090000	-1.1678780000
H	-4.4921770000	0.5038420000	-0.8378950000
H	-4.2669650000	1.8220500000	0.3256350000
H	-3.2161060000	0.9716740000	2.2748750000
H	-1.8721150000	-0.2876920000	2.1995460000
H	-1.3769020000	-1.9020370000	1.0283990000
H	-2.1574760000	-2.5890630000	-0.3585300000
H	0.0179080000	-3.4580860000	-0.4796890000
H	0.0073880000	-2.2027860000	-1.6840260000
H	1.6120830000	-2.5009730000	0.7881320000
H	3.3116660000	-1.3202010000	-0.5095650000
H	2.4810020000	-2.4544890000	-1.5674360000
H	2.0957070000	-0.7429510000	-1.6753840000

Hedycaryol carbocations:

A-OH

C	1.9179400000	2.2493050000	0.8608200000
C	2.0008290000	1.2696900000	-0.2316420000
C	3.2930080000	1.0152010000	-0.8757630000
C	3.7758510000	-0.3320180000	-0.2102970000
C	2.7066380000	-1.4020270000	-0.3814550000
C	1.3321590000	-0.9895070000	0.1578450000
C	1.3352530000	-0.8649470000	1.6835380000
C	0.8935160000	0.4219150000	-0.5930270000
C	-0.5207210000	0.8555240000	-0.2133880000
C	-1.5742420000	-0.2101750000	-0.5543650000
C	-2.9743070000	0.2466990000	-0.0710330000
C	-4.0268550000	-0.8371320000	-0.3124650000
C	-1.1811610000	-1.5691920000	0.0419330000
C	0.2491990000	-1.9770310000	-0.3058110000
H	2.8903310000	2.4658510000	1.3076500000
H	1.5700230000	3.1777910000	0.3708540000
H	1.1672080000	2.0027080000	1.6127730000
H	4.0216010000	1.8060960000	-0.6894220000
H	3.1815920000	0.8351390000	-1.9483450000
H	4.7093560000	-0.6084060000	-0.7058450000
H	4.0104590000	-0.1552950000	0.8435870000
H	2.6160690000	-1.6617380000	-1.4436240000
H	3.0171440000	-2.3143860000	0.1413900000
H	0.4079310000	-0.4340510000	2.0684820000
H	1.4247480000	-1.8684150000	2.1112870000
H	2.1703710000	-0.2723250000	2.0673970000
H	0.9608050000	0.1465300000	-1.6526410000
H	-0.5995810000	1.0822760000	0.8536070000
H	-0.7370470000	1.7827400000	-0.7542300000
H	-1.6272310000	-0.3114040000	-1.6491990000
H	-5.0200450000	-0.4560030000	-0.0540300000
H	-4.0523260000	-1.1354040000	-1.3656000000
H	-3.8362440000	-1.7191030000	0.3019560000
H	-1.3248700000	-1.5391570000	1.1278810000
H	-1.8476900000	-2.3464120000	-0.3403790000
H	0.4806940000	-2.9453130000	0.1555630000
H	0.3418300000	-2.1126380000	-1.3914850000
C	-3.4114350000	1.5439220000	-0.7577250000
H	-2.7606480000	2.3791790000	-0.4901540000
H	-3.4207070000	1.4312840000	-1.8466890000
H	-4.4263950000	1.8070670000	-0.4441560000
O	-2.8168660000	0.4718310000	1.3331180000
H	-3.6764820000	0.6673680000	1.7254320000

B-OH

C	-1.2576730000	2.3135500000	0.8694000000
C	-1.8101390000	1.2869660000	-0.0363900000
C	-3.2810080000	1.1276340000	-0.1456190000
C	-3.6874940000	-0.1863750000	-0.8147460000
C	-2.8308210000	-1.3656420000	-0.2959570000
C	-1.4383530000	-0.9406850000	0.2026910000
C	-1.4445290000	-0.7331350000	1.7250780000
C	-0.3319510000	-1.9339360000	-0.1856320000
C	1.0639840000	-1.5558710000	0.3225810000
C	1.3948440000	-0.0507680000	0.1990350000
C	2.9013380000	0.1739180000	-0.0817490000
C	3.7521300000	-0.4956360000	1.0002100000
C	3.2484230000	1.6640200000	-0.1561130000
C	0.5287270000	0.6105940000	-0.8864730000
C	-0.9795510000	0.3888190000	-0.7557420000
H	-0.2748810000	2.0430860000	1.2602940000
H	-1.9428970000	2.5862590000	1.6731760000
H	-1.1017550000	3.2062060000	0.2404700000
H	-3.6386930000	1.9924960000	-0.7296720000
H	-3.7247980000	1.2577260000	0.8491310000
H	-4.7491920000	-0.3692410000	-0.6404450000
H	-3.5711220000	-0.0939940000	-1.8982480000
H	-3.3447870000	-1.8725700000	0.5264320000
H	-2.7178030000	-2.0995060000	-1.1001320000
H	-1.5279900000	-1.7225050000	2.1860330000
H	-0.5300740000	-0.2736490000	2.1040020000
H	-2.3004990000	-0.1490120000	2.0764850000
H	-0.6281660000	-2.9077330000	0.2256440000
H	-0.3268160000	-2.0439390000	-1.2760840000
H	1.1775360000	-1.8716390000	1.3643490000
H	1.7919140000	-2.1228640000	-0.2644250000
H	1.2031450000	0.4405500000	1.1629610000
H	3.5869720000	-1.5745190000	1.0296640000
H	3.5321360000	-0.0765350000	1.9875390000
H	4.8143680000	-0.3235460000	0.7990050000
H	4.0703180000	-0.3995070000	-1.5642830000
H	4.3324830000	1.7916220000	-0.2355400000
H	2.7986640000	2.1345900000	-1.0331580000
H	0.7191460000	1.6874360000	-0.9246580000
H	0.8486110000	0.2087990000	-1.8526930000
H	-1.4343340000	0.0748890000	-1.6980640000
O	3.1310460000	-0.4421950000	-1.3471390000
H	2.9225910000	2.1939480000	0.7458840000

C-OH

C	2.1258060000	2.5542920000	-1.0517610000
C	1.9042730000	1.3364680000	-0.2612410000
C	2.5227040000	1.2291880000	1.0705810000
C	2.8141360000	-0.1944600000	1.5724070000
C	1.7117710000	-1.1776180000	1.1756930000
C	1.4001480000	-1.1404260000	-0.3308020000
C	2.6320420000	-1.5568070000	-1.1465850000
C	1.0201060000	0.2966440000	-0.7503910000
C	-0.3671060000	0.7800470000	-0.1281000000
C	-1.4893720000	-0.1985190000	-0.5190200000
C	-2.8201730000	0.2802520000	0.1266300000
C	-3.2635900000	1.6365490000	-0.4298480000
O	-2.5278710000	0.3984340000	1.5206260000
C	-3.9339440000	-0.7459980000	-0.0928510000
C	-1.1202550000	-1.6297650000	-0.1140060000
C	0.2286250000	-2.0646100000	-0.6869480000
H	2.3038780000	3.4384720000	-0.4348770000
H	3.0704760000	2.3525750000	-1.5899250000
H	1.3554660000	2.7244310000	-1.8055360000
H	1.7473750000	1.6995050000	1.7114190000
H	3.3811130000	1.9051660000	1.1450200000
H	3.7792450000	-0.5143050000	1.1693110000
H	2.9277370000	-0.1630640000	2.6581260000
H	0.8072740000	-0.9675640000	1.7570950000
H	2.0205290000	-2.1939620000	1.4426240000
H	2.4060220000	-1.5632150000	-2.2177070000
H	2.9524990000	-2.5650060000	-0.8666790000
H	3.4833570000	-0.8821040000	-0.9935730000
H	0.9010890000	0.3758830000	-1.8397170000
H	-0.3049990000	0.8199600000	0.9622110000
H	-0.5879550000	1.7857550000	-0.4948030000
H	-1.6234230000	-0.1597680000	-1.6100450000
H	-4.2271560000	1.9176400000	0.0058830000
H	-2.5542440000	2.4294260000	-0.1825700000
H	-3.3917630000	1.5950890000	-1.5161660000
H	-3.3455030000	0.5533880000	2.0092240000
H	-3.7315550000	-1.6712860000	0.4494120000
H	-4.0573290000	-0.9757110000	-1.1560420000
H	-4.8847450000	-0.3418600000	0.2695210000
H	-1.1345880000	-1.7043400000	0.9795050000
H	-1.8803800000	-2.3214190000	-0.4850960000
H	0.4696800000	-3.0800800000	-0.3518860000
H	0.1498410000	-2.1124910000	-1.7822920000

D-OH

C	-0.9971450000	0.3057990000	-0.7873080000
C	-1.6291170000	1.4662200000	-0.1467790000
C	-1.0082790000	2.7997880000	-0.2306650000
C	-2.9556180000	1.3844090000	0.4811690000
C	-3.4535570000	0.0103030000	0.9243220000
C	-2.9958920000	-1.0708260000	-0.0481940000
C	-1.4774800000	-1.0832340000	-0.2875130000
C	-1.1777200000	-2.1520070000	-1.3470100000
C	-0.7099910000	-1.3740670000	1.0163970000
C	0.8116850000	-1.3029750000	0.8744450000
C	1.2804240000	0.0555120000	0.3352770000
C	2.8122650000	0.1121760000	0.1069120000
C	3.5792360000	-0.3782870000	1.3368270000
C	3.2714100000	1.5312690000	-0.2448520000
C	0.5307420000	0.3919960000	-0.9676260000
H	-1.4564200000	0.4730770000	-1.7982340000
H	-1.6947830000	3.6039960000	0.0336450000
H	-0.1783680000	2.7928650000	0.4967290000
H	-0.5417300000	2.9790210000	-1.2027770000
H	-3.5890110000	1.7728060000	-0.3476110000
H	-3.0459950000	2.1649610000	1.2466440000
H	-3.0908740000	-0.1928440000	1.9355720000
H	-4.5437940000	0.0345600000	0.9872390000
H	-3.5065830000	-0.9364970000	-1.0125410000
H	-3.2999030000	-2.0541690000	0.3253360000
H	-1.5297210000	-3.1243010000	-0.9894020000
H	-1.7024440000	-1.9326720000	-2.2834530000
H	-0.1141980000	-2.2508720000	-1.5699980000
H	-1.0131560000	-0.6456650000	1.7844250000
H	-1.0092020000	-2.3608010000	1.3887850000
H	1.2514180000	-1.4827480000	1.8588220000
H	1.1831370000	-2.0942590000	0.2139440000
H	1.0466790000	0.8206690000	1.0961430000
H	3.2901250000	0.1790900000	2.2340220000
H	3.4077050000	-1.4422970000	1.5099300000
H	4.6537010000	-0.2296080000	1.1888310000
H	3.0141700000	2.2378310000	0.5519840000
H	4.3592130000	1.5531940000	-0.3616830000
H	0.8319860000	-0.3230250000	-1.7355880000
H	0.8196500000	1.3740840000	-1.3452820000
H	2.8381900000	1.8750890000	-1.1867950000
O	3.0428040000	-0.7622260000	-0.9971720000
H	3.9919970000	-0.8701260000	-1.1337250000

E-OH

C	3.1597150000	-1.7874300000	-0.1993710000
C	2.6089890000	-0.4253820000	0.2299660000
C	1.6112830000	0.1512110000	-0.8073940000
C	0.3456630000	-0.7318250000	-0.9360500000
C	-0.7939140000	-0.4176580000	0.0255590000
C	-1.7264700000	-1.5902210000	0.4331840000
C	-2.1141520000	-2.4869030000	-0.7413510000
C	-2.9378110000	-0.9019740000	1.0651550000
C	-3.2943010000	0.2231350000	0.0751240000
C	-1.9877140000	0.5889010000	-0.6349010000
C	-1.1898710000	1.6855110000	-0.2433000000
C	-1.4299770000	2.4130980000	1.0291360000
C	-0.0436340000	2.1354380000	-1.0820280000
C	1.2938120000	1.6242360000	-0.4843060000
C	3.7858070000	0.5248870000	0.4701740000
H	3.8648000000	-2.1524650000	0.5541180000
H	2.3785040000	-2.5422860000	-0.3052170000
H	3.7022280000	-1.7071020000	-1.1459910000
H	2.1208810000	0.1212390000	-1.7781200000
H	0.6287470000	-1.7690570000	-0.7414350000
H	-0.0238950000	-0.7209510000	-1.9674090000
H	-0.3832830000	-0.0129630000	0.9583540000
H	-1.1683050000	-2.1780690000	1.1721130000
H	-1.2463530000	-2.9773780000	-1.1881140000
H	-2.6320120000	-1.9343490000	-1.5329400000
H	-2.7914390000	-3.2709160000	-0.3929010000
H	-2.6617840000	-0.4841990000	2.0399110000
H	-3.7707810000	-1.5891380000	1.2300970000
H	-4.0038350000	-0.1273410000	-0.6773790000
H	-3.7547210000	1.0862690000	0.5620540000
H	-1.9540200000	0.3715850000	-1.7024770000
H	-2.0854570000	3.2564910000	0.7622930000
H	-1.9518460000	1.8168130000	1.7777030000
H	-0.5127500000	2.8345110000	1.4442960000
H	-0.1570230000	1.8112710000	-2.1197030000
H	-0.0373380000	3.2312620000	-1.0591350000
H	2.0897560000	2.2648770000	-0.8692400000
H	1.2753050000	1.7647320000	0.6020820000
H	3.4643630000	1.4664980000	0.9195940000
H	4.5056100000	0.0600850000	1.1512610000
H	4.3118760000	0.7403910000	-0.4653570000
O	1.8336600000	-0.5631470000	1.4289480000
H	2.3989490000	-0.8847860000	2.1423730000

F-OH

C	-2.1793050000	2.0888160000	0.8563620000
C	-2.2294940000	0.6950410000	0.2272160000
C	-1.2829590000	0.5747620000	-0.9891060000
C	0.1349330000	1.1514150000	-0.7769700000
C	0.9399570000	0.7231840000	0.4659780000
C	2.4288720000	1.1560150000	0.4000360000
C	2.7075540000	2.5152760000	-0.2306870000
C	3.1059620000	-0.0247990000	-0.2960360000
C	2.5266420000	-1.2615650000	0.3946900000
C	1.0347190000	-0.7852270000	0.8203970000
C	0.3082420000	-1.7954130000	0.0982890000
C	-0.1643710000	-3.0004530000	0.7977110000
C	0.0578150000	-1.6633000000	-1.3476950000
C	-1.2599480000	-0.8597430000	-1.5695650000
C	-3.6799870000	0.3960660000	-0.1644120000
H	-2.9021520000	2.1552450000	1.6757810000
H	-1.1948180000	2.3323440000	1.2576160000
H	-2.4502930000	2.8504250000	0.1187310000
H	-1.7373260000	1.2097970000	-1.7588640000
H	0.0477250000	2.2408300000	-0.7148120000
H	0.7118290000	0.9727790000	-1.6930140000
H	0.4595870000	1.1836180000	1.3325410000
H	2.7978660000	1.1784870000	1.4353930000
H	2.1503230000	3.3094610000	0.2754180000
H	2.4420340000	2.5342520000	-1.2917850000
H	3.7704500000	2.7569070000	-0.1519700000
H	4.1968110000	-0.0113350000	-0.2125540000
H	2.8665130000	-0.0225300000	-1.3675670000
H	2.5681710000	-2.1590250000	-0.2331310000
H	3.0410810000	-1.5041280000	1.3268080000
H	0.9217010000	-0.9315660000	1.8951000000
H	0.3333860000	-3.1599350000	1.7553360000
H	-1.2202650000	-2.7607360000	1.0147160000
H	-0.1465860000	-3.8950090000	0.1735270000
H	0.8827140000	-1.1283670000	-1.8240730000
H	-0.0515990000	-2.6439490000	-1.8154370000
H	-1.3817540000	-0.8262540000	-2.6552060000
H	-2.0854710000	-1.4568780000	-1.1776550000
H	-3.8042150000	-0.6035250000	-0.5854400000
H	-4.3239100000	0.4658750000	0.7185010000
H	-4.0426240000	1.1268280000	-0.8927560000
O	-1.7739260000	-0.2989390000	1.1668580000
H	-2.3495610000	-0.2825440000	1.9426790000

G-OH

C	-3.6966700000	-0.3770130000	1.0351280000
C	-2.6764180000	-0.0581600000	-0.0619620000
C	-1.4582020000	0.6848740000	0.5476840000
C	-0.5767950000	-0.2483260000	1.4117090000
C	0.7810230000	-0.8046720000	0.9467070000
C	0.9757790000	-1.8297410000	-0.1993400000
C	2.4719130000	-2.1423850000	-0.0089800000
C	3.1284080000	-0.7545240000	0.1655210000
C	1.9847080000	0.1507280000	0.7241690000
C	1.8233540000	1.4683150000	0.1281440000
C	2.9812370000	2.1313060000	-0.4932940000
C	0.5583660000	2.2294890000	0.1850590000
C	-0.6543300000	1.5024370000	-0.4648450000
C	-3.3578010000	0.7515300000	-1.1669660000
H	-4.5096790000	-0.9828010000	0.6216780000
H	-3.2488720000	-0.9375770000	1.8592270000
H	-4.1388580000	0.5398010000	1.4356410000
H	-1.8984660000	1.4209430000	1.2356450000
H	-1.1675730000	-1.1289010000	1.6764670000
H	-0.3735730000	0.2578340000	2.3649580000
H	2.9037850000	-2.6802100000	-0.8560530000
H	2.6256470000	-2.7518600000	0.8862160000
H	3.9933930000	-0.7793380000	0.8309010000
H	3.4731970000	-0.3834210000	-0.8026610000
H	3.9431230000	1.7011410000	-0.2178040000
H	2.8409340000	1.9664820000	-1.5788310000
H	2.9578100000	3.2135610000	-0.3485080000
H	0.3331990000	2.3916620000	1.2519370000
H	0.7091170000	3.2145180000	-0.2603850000
H	-1.2781750000	2.2947220000	-0.8848860000
H	-0.3237490000	0.8883820000	-1.3050640000
H	-2.7028320000	0.8675620000	-2.0344020000
H	-4.2638750000	0.2351750000	-1.4994630000
C	0.7362590000	-1.3484750000	-1.6306140000
H	1.0495690000	-2.1301600000	-2.3289550000
H	-0.3141810000	-1.1302270000	-1.8154890000
H	1.3396260000	-0.4603670000	-1.8736630000
H	0.3468300000	-2.7043640000	-0.0072630000
H	1.0974900000	-1.3755870000	1.8299890000
H	2.2946460000	0.5680240000	1.7207520000
H	-3.6584330000	1.7419270000	-0.8094800000
O	-2.1583810000	-1.2732250000	-0.6060540000
H	-2.8880390000	-1.8034200000	-0.9507800000

H-OH

C	-3.0166170000	-1.3789390000	0.5809210000
C	-2.4463470000	-0.1790100000	-0.1771200000
C	-1.5058930000	0.6831140000	0.7041240000
C	-0.3819720000	-0.0847430000	1.4586410000
C	1.0276950000	-0.2684650000	0.8589060000
C	1.6037940000	-1.7083840000	0.6903030000
C	2.8429030000	-1.5114010000	-0.1879170000
C	2.3587840000	-0.5996120000	-1.3276870000
C	1.2427870000	0.2590480000	-0.7312710000
C	1.5033880000	1.5099940000	-0.1299380000
C	2.8864490000	2.0117450000	0.1015520000
C	0.3849410000	2.4147880000	0.2756410000
C	-1.0204150000	1.9361650000	-0.0728190000
C	-3.6060290000	0.6775320000	-0.6971990000
H	-3.7778570000	-1.8746760000	-0.0293720000
H	-2.2467580000	-2.1186890000	0.8051840000
H	-3.4940140000	-1.0667400000	1.5149760000
H	-2.1584400000	1.0388240000	1.5100930000
H	-0.7545850000	-1.0761590000	1.7240180000
H	-0.2443800000	0.4278000000	2.4151370000
H	3.2445600000	-2.4590860000	-0.5531450000
H	3.6384010000	-1.0260380000	0.3890320000
H	3.1601200000	0.0064950000	-1.7583330000
H	1.9335390000	-1.1895770000	-2.1423470000
H	3.6310570000	1.2185210000	0.1607510000
H	3.1299230000	2.6247290000	-0.7786060000
H	2.9466400000	2.6605900000	0.9775040000
H	0.5007770000	2.6387020000	1.3459850000
H	0.5996470000	3.3706360000	-0.2259910000
H	-1.7079270000	2.7650490000	0.1128720000
H	-1.0625520000	1.7447040000	-1.1498630000
H	-3.2573080000	1.5057550000	-1.3173660000
H	-4.2771230000	0.0632480000	-1.3061800000
C	0.6096590000	-2.6921400000	0.0776480000
H	1.1296890000	-3.6196650000	-0.1772310000
H	-0.1847070000	-2.9527230000	0.7805080000
H	0.1356250000	-2.2942480000	-0.8239390000
H	1.8874760000	-2.0544280000	1.6914400000
H	1.7466340000	0.2249720000	1.5279480000
H	0.2577700000	0.1326870000	-1.1780480000
H	-4.1950120000	1.0816070000	0.1318240000
O	-1.6565020000	-0.6385310000	-1.2833270000
H	-2.2279400000	-1.0821920000	-1.9229460000

I-OH

C	1.9429860000	2.5813060000	0.9594350000
C	2.1198620000	1.4184090000	0.0891600000
C	3.4238620000	0.8450350000	-0.2683460000
C	3.1220860000	-0.3250050000	-1.2182030000
C	1.6393010000	-0.6902700000	-0.9647690000
C	1.0305900000	0.6702860000	-0.5138670000
C	-0.3532640000	0.8087350000	0.1289550000
C	-1.3952160000	-0.2389460000	-0.3089390000
C	-2.8151910000	0.3650190000	-0.1065820000
C	-3.1062810000	1.4264150000	-1.1742710000
C	-3.9254350000	-0.6866140000	-0.1542190000
C	-1.1715170000	-1.5564820000	0.4524440000
C	0.0056440000	-2.4117850000	-0.0296590000
C	1.4268640000	-1.8324280000	0.0446220000
C	1.8632880000	-1.4911570000	1.4724240000
H	2.8545770000	3.1602200000	1.1036230000
H	1.6215900000	2.1735810000	1.9343830000
H	1.1072860000	3.2071530000	0.6307250000
H	3.8498570000	0.5138470000	0.6963620000
H	4.1227030000	1.6114810000	-0.6263200000
H	3.7897630000	-1.1727970000	-1.0518190000
H	3.2617280000	0.0029070000	-2.2526110000
H	1.1700460000	-0.9949510000	-1.9048600000
H	-0.2601350000	0.7428570000	1.2186780000
H	-0.7275400000	1.8171850000	-0.0659540000
H	-1.2954810000	-0.4353890000	-1.3878600000
H	-4.0548830000	1.9249640000	-0.9527910000
H	-2.3350550000	2.1995810000	-1.2195460000
H	-3.1962080000	0.9653020000	-2.1626410000
H	-3.8488500000	-1.3076750000	-1.0525810000
H	-3.8999740000	-1.3308420000	0.7264710000
H	-1.0867370000	-1.3364140000	1.5247940000
H	-2.0541960000	-2.1900760000	0.3453290000
H	0.0003730000	-3.3419510000	0.5498120000
H	-0.1839270000	-2.7056130000	-1.0709880000
H	2.0854980000	-2.6393060000	-0.3055540000
H	1.3702520000	-0.5911940000	1.8625230000
H	1.6026450000	-2.3057640000	2.1536550000
H	2.9467080000	-1.3522400000	1.5457050000
H	1.0349490000	1.2803890000	-1.4624760000
H	-4.9010430000	-0.1902020000	-0.1805440000
O	-2.7702980000	0.9683610000	1.1891780000
H	-3.6570380000	1.2529540000	1.4419490000

J-OH

C	-1.3560760000	3.2261470000	-0.2948260000
C	-1.8122470000	1.8451520000	-0.1076820000
C	-3.2220590000	1.4118380000	-0.1334700000
C	-3.1783600000	-0.1270680000	-0.1472120000
C	-1.8718640000	-0.4355780000	0.6009280000
C	-0.9594710000	0.7064920000	0.1163660000
C	0.4698340000	0.8284980000	0.6088100000
C	1.3621780000	-0.3874610000	0.2295180000
C	2.8293760000	0.0965060000	0.0886300000
C	3.3037130000	0.7700660000	1.3812790000
C	3.7865430000	-1.0471200000	-0.2562120000
C	0.8558710000	-1.0951860000	-1.0399480000
C	-0.4430570000	-1.9491430000	-0.8992280000
C	-1.2473100000	-1.8264230000	0.4043570000
C	-2.3194940000	-2.9152550000	0.4938980000
H	-2.1096460000	3.8711220000	-0.7453550000
H	-0.4127860000	3.2612670000	-0.8495230000
H	-1.1153540000	3.6067830000	0.7117820000
H	-3.8285910000	1.9193200000	-0.8887680000
H	-3.5995060000	1.7641470000	0.8484270000
H	-3.1226880000	-0.4824960000	-1.1840010000
H	-4.0608640000	-0.5704670000	0.3142220000
H	-2.0467920000	-0.2786420000	1.6765620000
H	0.9255730000	1.7234880000	0.1719630000
H	0.4403960000	0.9677040000	1.6957270000
H	1.3614190000	-1.1034690000	1.0596700000
H	4.3406060000	1.1019240000	1.2699380000
H	2.7025690000	1.6486660000	1.6265650000
H	3.2688120000	0.0706820000	2.2228750000
H	4.8216940000	-0.6974970000	-0.1874150000
H	3.6746120000	-1.8806620000	0.4443990000
H	0.7531580000	-0.3416090000	-1.8316880000
H	1.6398030000	-1.7613060000	-1.4008290000
H	-1.1087780000	-1.7514870000	-1.7515250000
H	-0.1715960000	-3.0047950000	-0.9961240000
H	-0.5579020000	-1.9822600000	1.2423230000
H	-2.9976410000	-2.8765840000	-0.3656110000
H	-1.8599420000	-3.9063350000	0.5067330000
H	-2.9175590000	-2.8133670000	1.4050870000
H	-0.9229400000	0.5579000000	-1.0161320000
H	3.6281740000	-1.4133820000	-1.2716770000
O	2.7990150000	1.0524750000	-0.9758530000
H	3.6981940000	1.3427580000	-1.1720410000

K-OH

C	-3.2549740000	1.5411850000	-0.5203080000
C	-2.8423180000	0.2059310000	0.1036490000
C	-3.9369310000	-0.8263740000	-0.1722960000

O	-2.7300900000	0.4513220000	1.5070750000
C	-1.4512840000	-0.2681160000	-0.4318160000
C	-0.4460200000	0.8711630000	-0.1901470000
C	1.0637230000	0.5250870000	-0.3738770000
C	1.6613060000	-0.5012090000	0.6172450000
C	3.0811810000	0.0235860000	0.8970140000
C	2.9164550000	1.5475020000	0.7891490000
C	1.8736400000	1.7391600000	-0.2189090000
C	1.6158520000	3.0056610000	-0.9030340000
C	1.5588990000	-1.9681660000	0.1574100000
C	2.6779110000	-2.3963230000	-0.7951050000
C	0.1997540000	-2.2566140000	-0.4998650000
C	-1.0346640000	-1.6240210000	0.1621560000
H	-3.2007680000	1.5008450000	-1.6132780000
H	-4.2821210000	1.7749590000	-0.2329650000
H	-2.6278540000	2.3588400000	-0.1578140000
H	-4.9064680000	-0.4115780000	0.1144770000
H	-3.7901120000	-1.7512330000	0.3926030000
H	-3.9761880000	-1.0823230000	-1.2355060000
H	-3.0205340000	-0.3234690000	2.0017010000
H	-1.5549810000	-0.4078480000	-1.5184310000
H	-0.6939260000	1.6999540000	-0.8581560000
H	-0.5747310000	1.2218610000	0.8431000000
H	1.2218380000	0.2141950000	-1.4286370000
H	1.0791720000	-0.3935080000	1.5433910000
H	3.4694730000	-0.2855620000	1.8686520000
H	3.7875840000	-0.3068710000	0.1307240000
H	3.8055490000	2.1689420000	0.6583360000
H	2.4052150000	1.9233970000	1.7049620000
H	0.9411360000	3.5642040000	-0.2277770000
H	2.5167480000	3.6105140000	-1.0188270000
H	1.0793940000	2.8791250000	-1.8452060000
H	1.6327170000	-2.5825190000	1.0638000000
H	3.6546920000	-2.4374990000	-0.3071040000
H	2.4682320000	-3.3952850000	-1.1867680000
H	2.7533700000	-1.7228380000	-1.6591860000
H	0.0786530000	-3.3438240000	-0.5309660000
H	0.2396360000	-1.9429480000	-1.5542700000
H	-1.8678630000	-2.3152180000	0.0212090000
H	-0.8923200000	-1.5356010000	1.2477310000

L-OH

C	-1.9988120000	-2.4951490000	-1.1748210000
C	-1.8491880000	-1.4556370000	-0.1498400000
C	-2.9031120000	-1.0891370000	0.8098010000
C	-2.2975050000	0.0038840000	1.7073980000
C	-1.0843970000	0.5549140000	0.9227550000
C	-0.6694840000	-0.6497530000	0.0409380000
C	0.2995340000	-0.4505380000	-1.1232140000
C	1.5564500000	0.3670800000	-0.7742870000
C	2.5325030000	-0.3910880000	0.1582090000
C	2.9436890000	-1.7484720000	-0.4176760000
C	3.7823240000	0.4460270000	0.4319600000
C	1.2368730000	1.7921900000	-0.2483270000
C	-0.1238160000	2.3553330000	-0.6776110000
C	-1.3638260000	1.8284450000	0.1033110000
C	-2.5661660000	1.6952810000	-0.8344590000
H	-2.1169470000	-1.9667510000	-2.1355710000
H	-2.8623890000	-3.1397440000	-1.0145760000
H	-1.0794250000	-3.0806020000	-1.2799410000
H	-3.3276010000	-1.9676820000	1.3110830000
H	-3.7227020000	-0.7084910000	0.1726190000
H	-1.9621770000	-0.4450600000	2.6466620000
H	-3.0238220000	0.7800670000	1.9580100000
H	-0.2668610000	0.7604330000	1.6137660000
H	-0.1760440000	-1.3530310000	0.7783070000
H	0.5936990000	-1.4188060000	-1.5386410000
H	-0.2433440000	0.0667500000	-1.9222550000
H	2.0884090000	0.4798180000	-1.7282630000
H	2.0977020000	-2.4400920000	-0.4600580000
H	3.3640570000	-1.6426570000	-1.4226290000
H	3.7079830000	-2.2083060000	0.2162690000
H	4.4762970000	-0.1124450000	1.0690740000
H	4.3055510000	0.6760050000	-0.5011950000
H	1.3141340000	1.8067140000	0.8436760000
H	2.0163800000	2.4688440000	-0.6101400000
H	-0.0991910000	3.4434590000	-0.5678770000
H	-0.2645890000	2.1807870000	-1.7518790000
H	-1.6208110000	2.5855610000	0.8558610000
H	-3.5029290000	1.5227700000	-0.2951050000
H	-2.6948110000	2.6102100000	-1.4188960000
H	-2.4256960000	0.8789130000	-1.5596490000
H	3.5364230000	1.3827270000	0.9354460000
O	1.8115190000	-0.6039640000	1.3868010000
H	2.4261470000	-0.9079610000	2.0666620000
